# Comparative Plastid Phylogenomics of 29 Newly Sequenced *Bougainvillea* Cultivars Resolves the Phylogenetic Placement of Double-Bracted Cultivars

**DOI:** 10.3390/biology15201873

**Published:** 2026-10-17

**Authors:** Jiawei Yan, Xin Yang, Xia Lin, Xiaoyang Wang, Lina Sang, Ruikai Fu, Jun Yang, Xuan Chen, Chunyu Weng, Dingmin Liang, Lang Zhang, Huilin Zhao, Sai Huang, Junfang Ren, Jingfei Wang, Huiqi Zhao

**Affiliations:** 1 Sanya Institute, Hainan Academy of Agricultural Sciences, Sanya 572025, China; 25110710000002@hainanu.edu.cn (J.Y.); 18143710136@163.com (X.Y.); linxia23400@163.com (X.L.); 13075358645@163.com (X.W.); sanglina@foxmail.com (L.S.); juny83@126.com (J.Y.); renjunfang808@163.com (J.R.); 2 The Key Laboratory of Tropic Special Economic Plant Innovation and Utilization, and National Germplasm Resource Chengmai Observation and Experiment Station, Institute of Tropical Horticulture Research, Hainan Academy of Agricultural Sciences, Haikou 571100, China; fu0898@163.com (R.F.); chenxuan06@sina.com (X.C.); chunyuminmin@163.com (C.W.); m13307588828@163.com (D.L.); zlang123@126.com (L.Z.); huangsai512@163.com (S.H.); wangjf5821@163.com (J.W.); 3 School of Tropical Agriculture and Forestry, Hainan University, Haikou 571100, China; 4 Plant Protection and Quarantine Center of Shanxi Province, Taiyuan 030000, China; zhaohuilin666@163.com

**Keywords:** *Bougainvillea*, plastid genome, comparative genomics, phylogenomics, maternal lineage, double-bracted cultivars

## Abstract

*Bougainvillea* (Nyctaginaceae) cultivars are horticulturally prized worldwide for their vivid bracts; however, repeated interspecific hybridization and somatic mutations have obscured their maternal origins, particularly for double-bracted forms. Here, we sequenced and assembled the complete plastomes of 29 cultivars (16 double-bracted, 13 single-bracted) and conducted comparative and phylogenomic analyses alongside 15 published taxa. All plastomes displayed a conserved quadripartite structure (153,994–154,542 bp, comprising 130–131 functional genes), with *B.* × *buttiana* ‘Mahara Variegated’ harboring a pseudogenized *ycf1* caused by a verified frameshift-induced premature stop codon, serving as a unique diagnostic marker for this cultivar. Sequence divergence was largely confined to intergenic spacers, identifying *trnH-GUG–psbA* and *trnS-GCU–trnG-UCC* as potential DNA barcodes for species-level discrimination. Conversely, intraspecific variation was virtually absent; 13 double-bracted accessions were completely sequence-identical. Phylogenomic analyses resolved interspecific backbones (*B. spectabilis*, *B. peruviana*, and *B. glabra*) well but placed nearly all double-bracted cultivars into an unresolved polytomy alongside single-bracted *B.* × *buttiana* lineages (including ‘Mrs. Butt’). This confirms a shared maternal origin and demonstrates that double-bracted phenotypes predominantly arose through recurrent somatic bud mutations rather than independent hybridizations. Furthermore, identical plastomes across distinct phenotypes highlight the inherent resolution limits of maternal markers for cultivar delimitation. Overall, our study enriches *Bougainvillea* genomic resources, clarifies previously ambiguous maternal lineages among cultivated taxa, and demonstrates that cultivar-level discrimination must rely on nuclear rather than plastid genomic markers.

## Simple Summary

*Bougainvillea* is a popular tropical ornamental plant, but extensive hybridization and natural mutations have obscured the origins of its diverse cultivars, particularly showy double-bracted varieties. Here, we sequenced the complete chloroplast genomes of 29 cultivars to resolve their maternal relationships. We found that most double-bracted cultivars share identical chloroplast sequences tracing back to a common maternal ancestor (*B.* × *buttiana*), demonstrating that they arose mainly through natural branch mutations (bud sports) rather than separate cross-breeding events. These findings clarify cultivar classification and provide valuable genomic resources to guide parental selection and breeding for urban greening.

## 1. Introduction

*Bougainvillea* Comm. ex Juss. (Nyctaginaceae) is among the most widely grown ornamental crops of warm climates, valued for the range of its bract colours, its hardiness, and its capacity to tolerate pollution. The genus is South American in origin and comprises 18 species [1,2,3]. Three of them—*B. spectabilis* Willd., *B. glabra* Choisy, and *B. peruviana* Humb. & Bonpl.—account for most of the horticultural interest, and it is their repeated crossing that has produced the modern cultivars [1]. Those cultivars are usually placed in three hybrid groups. The first goes back to a deep-red plant that Mrs. R. V. Butt found at Cartagena, Colombia, early in the twentieth century; a cross between a form of *B. glabra* and a pink-bracted *B. peruviana*, it was named *B.* × *buttiana* ‘Mrs. Butt’ after her [1,4]. Almost every later colour variant arose as a bud sport of ‘Mrs. Butt’, and the whole assemblage was eventually treated as the nothospecies *B.* × *buttiana* [1,5]. The second group, *B.* × *specto-peruviana*, comprises hybrids of *B. spectabilis* and *B. peruviana* whose young coppery-red bracts age to magenta or pink [1,5,6]. The third, *B.* × *specto-glabra*, comprises hybrids of *B. spectabilis* and *B. glabra* with pale-purple to purple bracts [1,5,7]. From these beginnings, more than 500 cultivars have been raised by natural and controlled crossing and by spontaneous and induced mutation, differing in leaf form, foliage colour, and the shape and pigmentation of the bracts [8].

By bract morphology the cultivars divide into single-bracted forms, with one whorl of bracts, and double- or multi-bracted forms, with several overlapping whorls [1,5]. Variegation is another conspicuous trait, and variegated cultivars are generally taken to be mutants of existing single- or double-bracted stock. Single-bracted cultivars are by far the more numerous; the double-bracted group began with only four—‘Cherry Blossom’, ‘Los Banos Beauty’, ‘Mahara’, and ‘Rose Ville Delight’ [9,10]—and has since grown, through further mutation, to more than 20 on record, among them ‘Carson’s Gold’, ‘Dania White’, ‘Mrs. Eusenia Raja Singh’, ‘Mahara Pink’, ‘Mahara Ice Cream’, ‘SF Marble’, ‘Aussie Gold Splash’, ‘SF Ruby Mahara’, ‘Marietta’, ‘Mahara Variegated’, ‘Lizard Double Orange’, ‘Pallavi’, ‘Cinderella’, ‘Double White Var.’, ‘Mahara Dewi’, and ‘Aussie Red Batik’. The recorded histories of a few illustrate how the group has grown: ‘Cherry Blossom’ is a bud sport of ‘Los Banos Beauty’ and is associated with *B.* × *buttiana* [9]; ‘Los Banos Beauty’ is itself said to derive from *B. glabra* [10]; and ‘Mahara’ and ‘Rose Ville Delight’ arose as bud sports of the *B.* × *buttiana* cultivars ‘Mrs. Butt’ and ‘Mrs. McClean’ [10]. Even so, the single-bracted progenitors of most double-bracted cultivars, and their relationships to one another, are still not settled.

Plastid genomic research in *Bougainvillea* has advanced rapidly. The plastome is now known to be a conserved quadripartite molecule of about 154 kb [6,11,12,13,14], and comparative work on *B. spectabilis*, *B. glabra*, and *B. peruviana* has described its gene content, codon usage, and IR-boundary behaviour [15]. That work has also flagged hypervariable regions such as *trnH-GUG–psbA* and *ycf1*, together with SSR loci, as candidate markers—tools that matter because hybridization and phenotypic convergence make many cultivars hard to tell apart [14,16]. On the phylogenetic side, Bautista et al. [13] placed *B. pachyphylla* Heimerl ex Standl. and *B. peruviana* near the base of the genus, with *B. spinosa* (Cav.) Heimerl sister to a clade of cultivated species (among them *B. spectabilis* and *B. glabra*) and a clade of wild ones (such as *B. berberidifolia* Heimerl), a pattern consistent with early divergence; however, that study aimed at species-level divergence and included no double-bracted cultivars at all. A later study of South Chinese cultivars [17] recovered the double-bracted ‘Carson’s Gold’ (syn. ‘Double Yellow’) and ‘Rose Ville Delight’ (syn. ‘Double Salmon’) in the same clade as several single-bracted *B.* × *buttiana* cultivars, *B. glabra* ‘White Stripe’, and *B. spectabilis* ‘Splendens’. Nevertheless, Wu et al. [17] sampled only three double-bracted cultivars in total. Because Bautista et al. lacked double-bracted taxa entirely and Wu et al. surveyed too few to represent the group’s phenotypic diversity, critical evolutionary questions—such as whether double-bracted forms arose from a single mutational event or multiple independent origins, and which specific single-bracted forms gave rise to them—remain completely open. Resolving these questions requires a targeted strategy that couples dense sampling of double-bracted cultivars with representative single-bracted progenitors.

Since double-bracted cultivars are maintained almost entirely by vegetative cuttings, their maternally inherited plastomes offer a stable record of their maternal ancestry. Although somatic mutations can theoretically accumulate in plastomes during prolonged asexual propagation, the inherently low mutation rate of plant plastid genomes yields at most a few sporadic variants over horticultural timescales [18,19]. In contrast, interspecific hybridization and maternal replacement involve substantial, fixed sequence divergence accumulated over extensive evolutionary divergence among ancestral species (*B. spectabilis*, *B. glabra*, and *B. peruviana*). Therefore, whole-plastome sequences can readily distinguish deep maternal lineages from intra-cultivar somatic drift. Because conflicting historical records and earlier fragmented markers assigned morphologically divergent double-bracted cultivars to distinct species backgrounds, we formulated two testable hypotheses: (1) whether double-bracted *Bougainvillea* cultivars originated through multiple independent events across distinct hybrid lineages (as suggested by conflicting historical records) or share a single maternal background; and (2) whether complete plastomes provide sufficient phylogenetic resolution to clarify these maternal hybrid lineages and determine the extent to which plastomic variation can resolve cultivar-level relationships versus reflecting clonal stasis.

**Figure 1:**
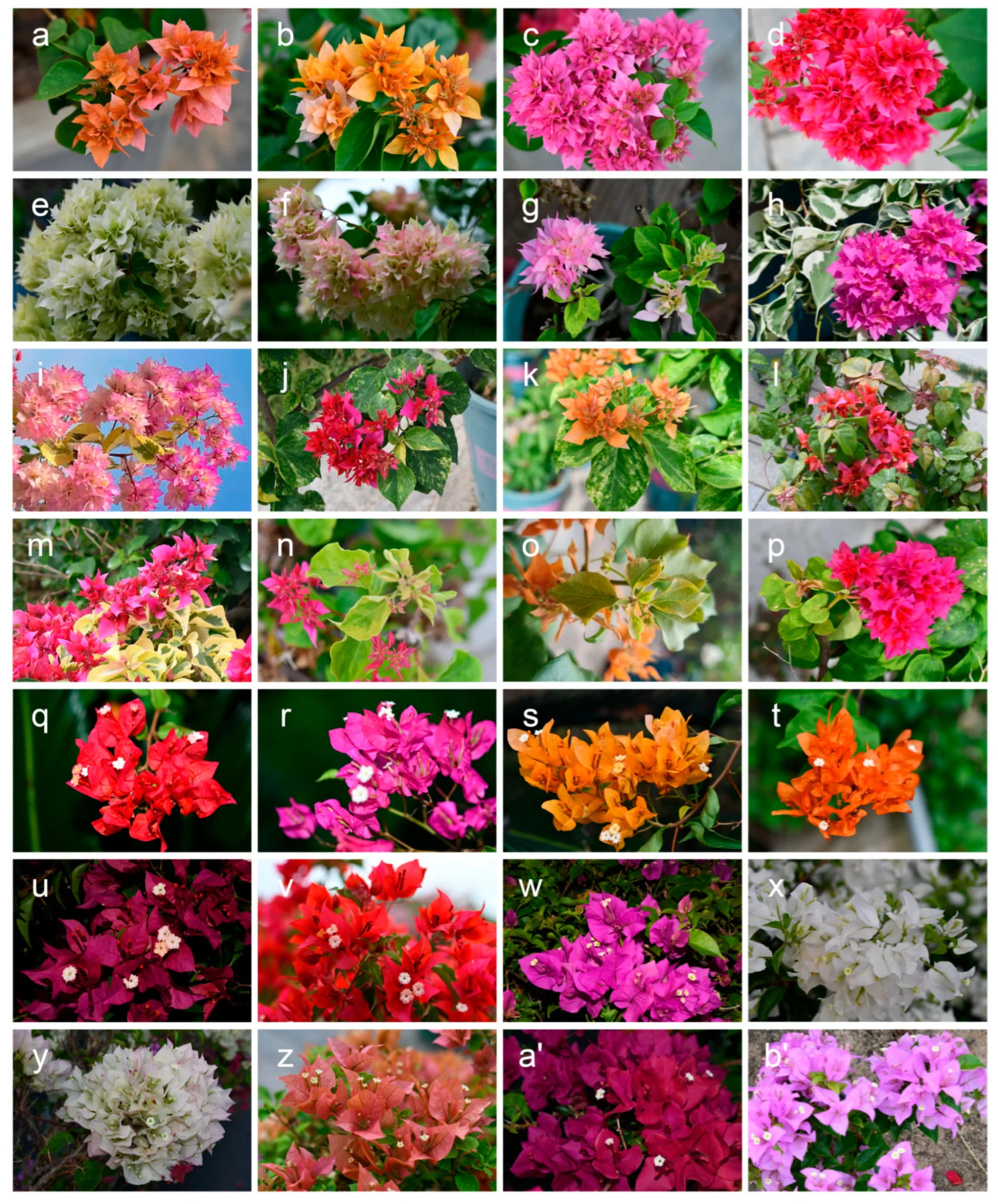
Morphological features of the representative *Bougainvillea* cultivars used for plastome sequencing. (**a**) ‘Rose Ville Delight’; (**b**) ‘Carson’s Gold’; (**c**) ‘Los Banos Beauty’; (**d**) ‘Mahara’; (**e**) ‘Dania White’; (**f**) ‘Cherry Blossom’; (**g**) ‘Mrs. Eusenia Raja Singh’; (**h**) ‘Mahara Pink’; (**i**) ‘Mahara Ice Cream’; (**j**) ‘SF Marble’; (**k**) ‘Aussie Gold Splash’; (**l**) ‘SF Ruby Mahara’; (**m**) ‘Marietta’; (**n**) ‘Mahara Variegated’; (**o**) ‘Lizard Double Orange’; (**p**) ‘Cinderella’; (**q**) ‘Chili Red’; (**r**) ‘Chili Purple’; (**s**) ‘Chili Yellow’; (**t**) ‘Chili Orange’; (**u**) ‘Ruby’; (**v**) ‘Flame’; (**w**) ‘Elizabeth Angus’; (**x**) ‘Mrs. Eva White’; (**y**) ‘Audrey Grey’; (**z**) ‘Pedro’; (**a′**) ‘Barbara Karst’; (**b′**) ‘Mrs. Eva’. Due to space limitations, ‘Mrs. Butt’ is not shown here; readers are referred to Lan et al. [4] for its photographs. Photo credit: All photographs were taken by the authors.

Testing these hypotheses within a genus of over 500 recognized cultivars requires a targeted sampling strategy that maximizes morphological diversity while minimizing clonal bias. We therefore selected 16 double-bracted cultivars—including ‘Mahara’, ‘Carson’s Gold’, ‘Rose Ville Delight’, and ‘Los Banos Beauty’ (Figure 1a–p)—encompassing nearly the entire phenotypic breadth of known double-bracted forms. To establish an evolutionary reference framework, these were paired with 13 representative single-bracted cultivars (Figure 1q–b’, plus ‘Mrs. Butt’) that span the three primary hybrid groups and represent the core morphological diversity of *Bougainvillea* (encompassing the full spectrum of bract colors and foliage variegation). We sequenced and assembled complete plastomes for these 29 cultivars to reconstruct their phylogenomic framework, pinpoint maternal lineages, and quantify structural and sequence variation across the genus. This study provides a robust maternal phylogenetic baseline for *Bougainvillea*, resolves longstanding debates regarding the origins of double-bracted forms, and critically evaluates the utility and boundaries of plastid markers in cultivar identification and germplasm management.

## 2. Material and Methods

### 2.1. Taxon Sampling, DNA Extraction and Sequencing

We selected 16 double-bracted and 13 single-bracted cultivars covering the main cultivated hybrid groups of *Bougainvillea*, and drew a further 15 published plastomes of *Bougainvillea* and related taxa from GenBank; sample details and accession numbers are in Supplementary Data set S1. Total genomic DNA was extracted from fresh leaves by a modified CTAB method [20], and its integrity, purity, and concentration checked by agarose gel electrophoresis, NanoDrop spectrophotometer (Thermo Fisher Scientific, Waltham, MA, USA), and Qubit fluorometer (Thermo Fisher Scientific, Waltham, MA, USA). DNA that passed was sheared on a Covaris ultrasonicator (Covaris, Woburn, MA, USA), and paired-end libraries were made by end repair, A-tailing, adapter ligation, purification, and PCR. Libraries were checked on Qubit, an Agilent 2100 Bioanalyzer (Agilent Technologies, Santa Clara, CA, USA), and by qPCR, and those that passed were sequenced on a DNBSEQ-T7 platform (MGI Tech Co., Ltd., Shenzhen, China).

### 2.2. Plastome Assembly and Annotation

Raw reads were processed using fastp v0.23.4 [21] to remove adapters and filter low-quality reads and reads containing excessive ambiguous bases. Plastomes were assembled using NOVOPlasty v4.3.1 [22], with CAP3 used to join contigs when necessary [23]. Assemblies were manually inspected and standardized to a common starting position and orientation. Genome annotation was performed using GeSeq [24], with tRNA genes checked using tRNAscan-SE v2.0.7 [25]. Annotations were manually curated against the plastomes of *B. peruviana* (NC_049005), *B. pachyphylla* (NC_056255), and *B. stipitata* (NC_065347).

Plastome length, regional lengths, GC content, gene content, and intron organization were summarized for the 29 newly assembled plastomes. Circular genome maps and gene-splicing structures were visualized using Chloroplot [26] and CPGView [27], respectively. Gene order and orientation were compared using aligned plastomes and annotation tracks in Geneious Prime v2025.0.2 [28] to assess collinearity and identify potential structural rearrangements.

### 2.3. Read-Mapping Validation and Sequencing-Quality Assessment

Quality-filtered paired-end reads were mapped to their corresponding final plastome assemblies using Bowtie 2 v2.5.4 [29]. Alignments were sorted and indexed using SAMtools v1.21 [30], and primary mapped alignments were retained without an additional mapping-quality threshold.

Per-base sequencing depth was calculated using SAMtools depth, including zero-coverage positions. Mean, median, and minimum depth, the number of uncovered positions, and the proportions of sites covered at ≥1×, ≥10×, ≥30×, and ≥100× were summarized for each accession. Per-site mean Phred scores were calculated from SAMtools mpileup output without additional mapping- or base-quality filtering and with base-alignment-quality adjustment disabled. Overall mean base quality and the proportion of evaluable positions with mean quality ≥Q30 were summarized. Depth and mean-quality profiles were averaged within non-overlapping 200 bp windows, with depth plotted as log_10_(mean depth + 1). Visualizations were generated using Matplotlib v3.10.6 [31] in Python v3.12.10.

### 2.4. Validation of the Putative ycf1 Pseudogene

Read support for the putative *ycf1* pseudogene in *B.* × *buttiana* ‘Mahara Variegated’ was examined across plastome positions 124,870–130,528 (5659 bp). Per-base depth was calculated using SAMtools depth with a minimum base quality of 20 and no additional mapping-quality threshold, and plotted as log_10_(depth + 1). Nucleotide and insertion/deletion (InDel) support was obtained using SAMtools mpileup with base-alignment-quality adjustment disabled and a maximum depth of 100,000. A custom Python script summarized nucleotide depth (A + C + G + T), major-allele and combined non-major-allele frequencies, support for the most-supported InDel, and mean base quality.

The *ycf1* region was additionally examined by PCR amplification, molecular cloning, and Sanger sequencing. The cloned sequence was compared with the assembly-derived sequence and representative *Bougainvillea ycf1* homologs. Nucleotide and translated amino-acid sequences were aligned separately using MAFFT v7.505 [32]. Amino-acid identity relative to the intact *B.* × *buttiana* reference sequence (OR344376) was calculated using 30-aa sliding windows with a step size of 1 aa. Reference-gap columns were excluded, whereas query gaps were counted as mismatches. Nucleotide alignments and translations retaining internal stop codons were examined for concordant frameshifts and premature termination between the cloned and assembly-derived sequences.

### 2.5. Phylogenetic Analysis

Phylogenetic relationships were reconstructed from 44 plastomes using complete-plastome and concatenated coding-sequence matrices. Complete plastome sequences were aligned using MAFFT v7.505 [32]. Both the original alignment and an alignment processed using the automated1 mode in trimAl v1.4 [33] were retained. Homologous protein-coding sequences shared among the 44 plastomes were also extracted, aligned with MAFFT and concatenated into a CDS matrix. Maximum-likelihood analyses were performed independently for each matrix using IQ-TREE v3.1.1 [34]. ModelFinder selected the best-fitting nucleotide substitution model according to the Bayesian information criterion [35]. The TVM + F + I + R4 model was selected for the 165,284 bp untrimmed complete-plastome alignment, K3Pu + F + I + R4 for the 150,726 bp trimmed complete-plastome alignment, and GTR + F + I + G4 for the 69,420 bp concatenated CDS matrix. Branch support was assessed using 1000 ultrafast bootstrap and 1000 SH-aLRT replicates [36,37].

Bayesian analyses of the same matrices were conducted independently using MrBayes v3.2 [38], with a six-rate nucleotide substitution model, gamma-distributed among-site rate variation and equal base frequencies (nst = 6, rates = gamma and statefreqpr = fixed(equal)). Two independent runs, each comprising four Markov chains, were conducted for 6,000,000 generations, with sampling every 100 generations. The first 25% of samples were discarded as burn-in, and posterior probabilities were calculated from the remaining trees. *Seguieria aculeata* Jacq., *Monococcus echinophorus* F.Muell., *Acleisanthes obtusa* (Choisy) Standl. and *Nyctaginia capitata* Choisy were used as outgroups.

### 2.6. Pairwise Sequence Variation and Plastid Haplotype Analysis

The 29 newly assembled plastomes were aligned using MAFFT. Pairwise sequence identities and SNP and InDel differences were calculated using Geneious Prime v2025.0.2 and summarized as pairwise matrices. SNP and InDel counts were transformed as log_10_(count + 1) for heatmap visualization.

Plastid haplotypes were identified using DnaSP v6.12 [39], excluding alignment columns containing gaps or missing data. The resulting haplotype data were imported into PopART v1.7 [40] to construct a TCS network [41]. Single- and double-bracted phenotypes were mapped onto the network. Node sizes represented haplotype frequencies, and branch labels indicated mutational steps.

### 2.7. Comparative Plastome Analysis, Nucleotide Diversity, and IR Boundaries

Seventeen representative plastomes were selected to cover the major phylogenetic lineages, including seven cultivars from the *B.* × *buttiana* clade. Sequence conservation across coding and non-coding regions was examined using mVISTA, with ‘Mahara’ as the reference [42].

Nucleotide diversity (Pi) was calculated using DnaSP with a 500 bp sliding window and a 50 bp step size for the 17 representative plastomes and, separately, the *B.* × *buttiana* subset. Windows were ranked by Pi, and high-diversity regions were annotated against the reference plastome to identify candidate marker regions. The LSC/IRb, IRb/SSC, SSC/IRa, and IRa/LSC junctions of the 17 plastomes were compared using IRscope [43] to characterize boundary variation and associated gene positions.

## 3. Results

### 3.1. Structural Features of Plastid Genomes

**Figure 2:**
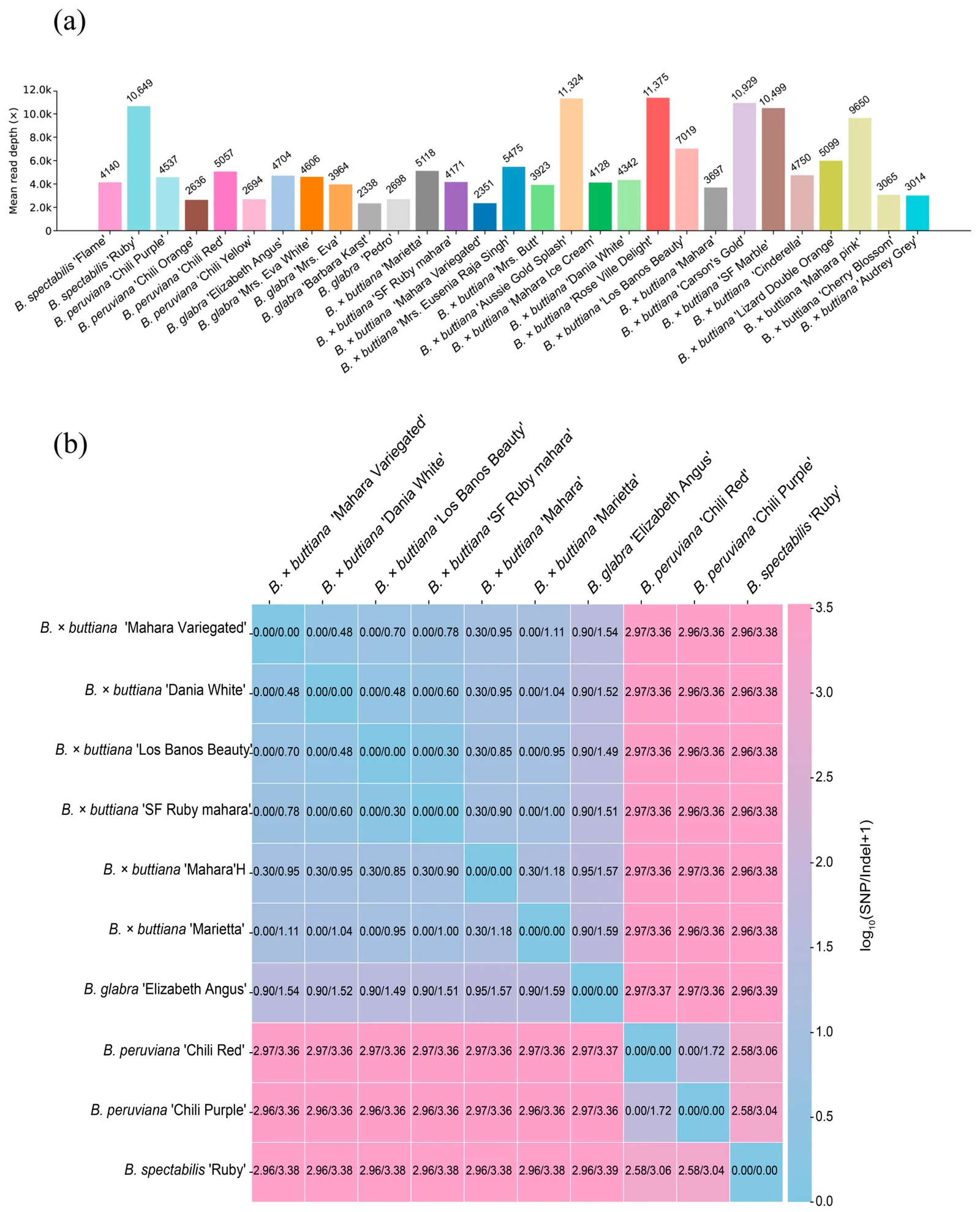
Plastome sequencing depth and pairwise sequence divergence of *Bougainvillea* cultivars. (**a**) Mean sequencing depth across complete plastomes for the 29 newly sequenced cultivars, with exact average depth values indicated above the bars. (**b**) Heatmap of pairwise SNP and InDel divergence among 10 representative cultivars (complete pairwise dataset for all 29 cultivars is provided in Supplementary Data set S6). Values within cells represent Log10 (SNP + 1)/Log10 (InDel + 1), with the color gradient from blue to pink indicating increasing sequence divergence.

To characterize the plastome structural features of the 29 new cultivars, we first evaluated their sequencing quality. The sequencing depth ranged from 2338× to 11,375×, with the average base quality exceeding Q30 for all samples, indicating high data quality that fully meets the requirements for high-precision assembly (Figure 2a; Supplementary Data set S2; Supplementary Figures S1 and S2). Based on these high-quality data, we successfully assembled 29 complete plastomes. All 29 newly assembled plastomes displayed the typical quadripartite structure, comprising a large single-copy (LSC) region, a small single-copy (SSC) region, and a pair of inverted repeats (IRa and IRb), as exemplified by the circular map of ‘Mahara’ (Supplementary Figure S3). Pairwise sequence identity among the 29 plastomes ranged from 97.27% to 100% (Supplementary Data set S3), reflecting high overall sequence conservation. Multiple sequence alignment demonstrated that gene content and order were strictly collinear across all plastomes, with no structural rearrangements (such as inversions or translocations) detected (Supplementary Figure S4).

Genome length ran from 153,994 to 154,542 bp (Supplementary Data set S4). The commonest length, 154,542 bp, was observed in 16 cultivars, although these were not entirely sequence-identical: 13 *B*. × *buttiana* cultivars shared 100% pairwise sequence identity, whereas the other three shared the same length but differed by minor single-nucleotide polymorphisms (SNPs) (Supplementary Tables S3 and S6; Supplementary Figures S5 and S6); the rest measured 153,994 bp (2 cultivars), 154,415 bp (1), 154,465 bp (3), 154,520 bp (5), 154,534 bp (1), and 154,541 bp (1) (Supplementary Table S4). Most of this variation lay in the LSC region (85,170–85,702 bp), the IR (25,377–25,426 bp) and SSC (18,043–18,084 bp) regions being more stable. GC content varied little among cultivars, whether measured across the whole genome (36.5–36.6%) or within the LSC (34.2–34.3%), SSC (29.5%), or IR (42.8%) regions. Several cultivars were sequence-identical: ‘Flame’ with ‘Ruby’, ‘Chili Orange’ with ‘Chili Yellow’, the five-cultivar set ‘Elizabeth Angus’, ‘Mrs. Eva White’, ‘Mrs. Eva’, ‘Barbara Karst’, and ‘Pedro’, and a 13-cultivar *B.* × *buttiana* set (‘Audrey Grey’, ‘Cherry Blossom’, ‘Mahara Pink’, ‘Lizard Double Orange’, ‘Cinderella’, ‘SF Marble’, ‘Carson’s Gold’, ‘Los Banos Beauty’, ‘Rose Ville Delight’, ‘Mahara Ice Cream’, ‘Aussie Gold Splash’, ‘Mrs. Butt’, and ‘Mrs. Eusenia Raja Singh’) (Supplementary Data set S4).

Each plastome carried 130–131 functional genes: 85–86 protein-coding genes (PCGs), 37 tRNAs, and 8 rRNAs. The LSC region held 85 genes (63 PCGs and 22 tRNAs) and the SSC region 13–14 (12–13 PCGs and 1 tRNA), with *ycf1* pseudogenized in ‘Mahara Variegated’ owing to a frameshift mutation that introduced a premature stop codon and severely truncated its open reading frame (Supplementary Figure S7). Notably, high-depth read mapping across this region (mean regional depth = 2093.7×; depth at the mutation locus = 2045×) revealed that 99.85% of the mapped reads supported this frameshift at codon 458. This was further confirmed by molecular cloning and Sanger sequencing, unambiguously demonstrating that this is an authentic, fixed homoplasmic mutation rather than a sequencing artifact, plastome heteroplasmy, or chimerism (Supplementary Data set S5; Supplementary Figure S7). Eighteen genes lying within the IRs were present in two copies—7 PCGs (*ndhB*, *rpl2*, *rpl23*, *rps7*, *rps12*, *ycf1*, and *ycf2*), 7 tRNAs (*trnA-UGC*, *trnI-CAU*, *trnI-GAU*, *trnL-CAA*, *trnN-GUU*, *trnR-ACG*, and *trnV-GAC*), and 4 rRNAs (*rrn16*, *rrn23*, *rrn4.5*, and *rrn5*) (Table 1). Fourteen genes contained a single intron—8 PCGs (*rps16*, *atpF*, *rpoC1*, *petB*, *petD*, *rpl16*, *ndhB*, and *ndhA*) and 6 tRNAs (*trnK-UUU*, *trnG-UCC*, *trnL-UAA*, *trnV-UAC*, *trnI-GAU*, and *trnA-UGC*)—and three (*pafI*, *rps12*, and *clpP1*) contained two. In every plastome *rps19* sat across the LSC/IR junction, and *ycf1* and *ndhF* across the IR/SSC junction. *rps12* was trans-spliced, its 5′ exon in the LSC and its two duplicated 3′ exons in IRa and IRb; all the other intron-bearing PCGs (*rps16*, *atpF*, *rpoC1*, *pafI*, *clpP1*, *petB*, *petD*, *rpl16*, *ndhB*, and *ndhA*) were cis-spliced (Supplementary Figure S8).

### 3.2. Phylogenetic Relationships

**Figure 3:**
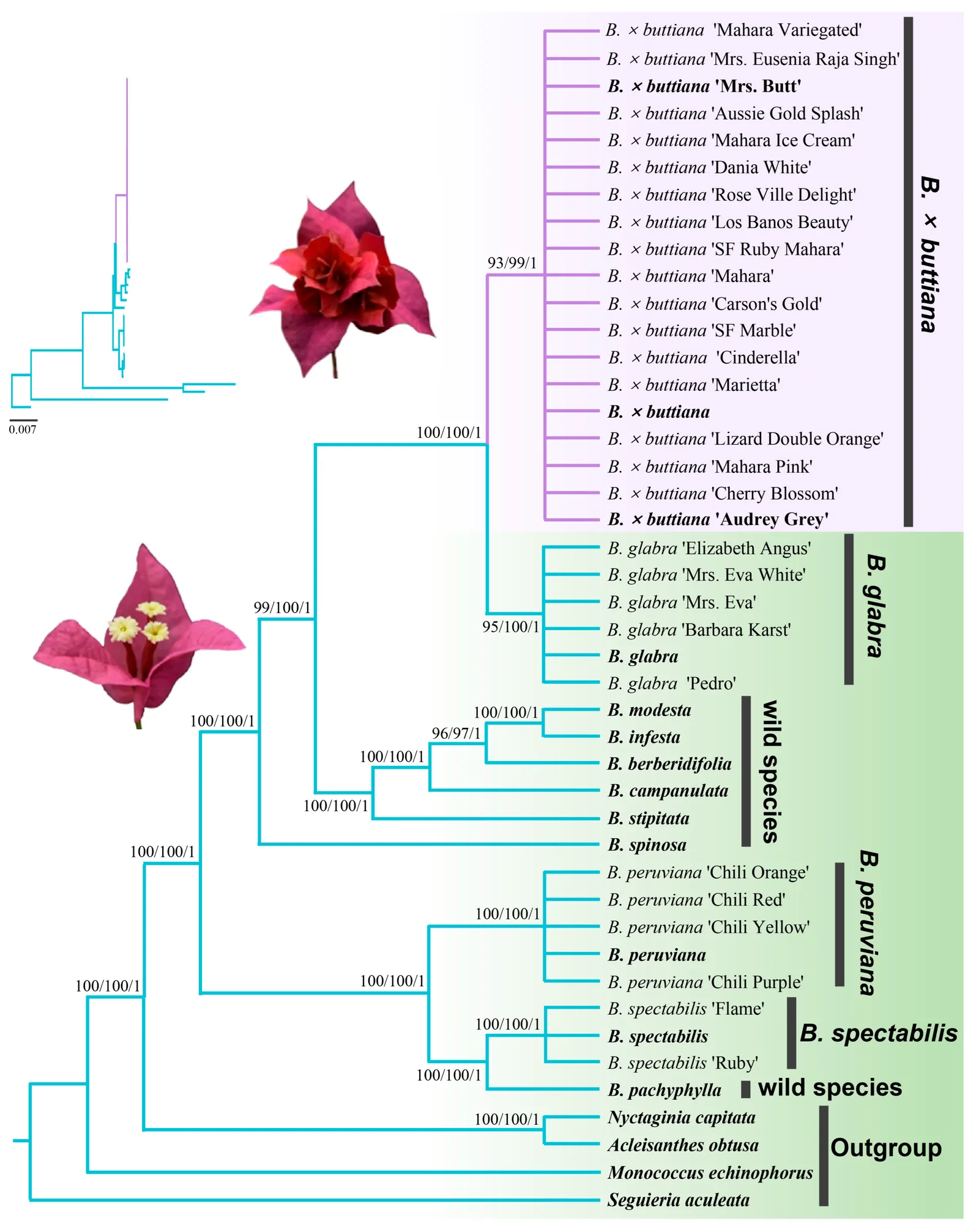
Phylogenetic relationships inferred from Maximum likelihood (ML)and Bayesian inference (BI) based on 44 complete plastid genomes, rooted with outgroups from Nyctaginaceae and Petiveriaceae (Caryophyllales). Numbers at nodes indicate SH-aLRT support (%), ultrafast bootstrap support (UFBoot, %), and Bayesian posterior probabilities (BPP), respectively; values below 50% (or 0.50 for BPP) are omitted. Accessions retrieved from NCBI are shown in bold black font. Within the buttiana clade, single-bracted cultivars are highlighted in bold. For visual clarity, the main tree is displayed as a cladogram; the inset in the upper-left corner presents the identical topology as a phylogram, with the scale bar representing 0.007 nucleotide substitutions per site.

We reconstructed phylogenetic trees using ML and BI based on untrimmed complete plastomes, trimmed plastomes, and protein-coding sequences (CDS) across 44 accessions to assess the phylogenetic placement of the newly sequenced taxa and examine relationships between double- and single-bracted cultivars. The initial MAFFT alignment of the complete plastomes was 165,284 bp in length and contained 15,704 variable sites, including 7269 parsimony-informative sites. After filtering with trimAl using the -automated1 option, the alignment was reduced to 150,726 bp, with 14,558 positions (8.81%) removed. The trimmed alignment retained 13,743 variable sites, including 6701 parsimony-informative sites. Comparative evaluations revealed that the untrimmed complete plastomes best resolved these cultivars (Figure 3). In comparison, the trimmed dataset recovered a consistent topology, albeit with slightly lower support values, while the CDS dataset lacked sufficient variation to differentiate these accessions (Supplementary Figures S9–S11). Within *Bougainvillea*, the species were divided into two well-supported primary clades (both with 100/100/1 support). The first clade (Clade I) comprises *B. pachyphylla*, *B. spectabilis* and *B. peruviana*. At the base of this clade, *B. pachyphylla* was resolved as sister to a group (100/100/1) that further split into two sister subclades: one containing *B. spectabilis* and its cultivars (‘Flame’ and ‘Ruby’), and the other comprising wild *B. peruviana* and the four ‘Chili’ cultivars (‘Chili Orange’, ‘Chili Red’, ‘Chili Yellow’ and ‘Chili Purple’). The second clade (Clade II) encompasses the remaining wild and cultivated taxa. Within this clade, *B. spinosa* was sister to all other members (100/100/1). The remaining taxa then diverged into a wild species lineage and a cultivated species lineage (99/100/1). The wild species formed a fully supported group (100/100/1), diverging successively in the order of *B. stipitata*, *B. campanulata*, *B. berberidifolia* and the sister pair *B. infesta* and *B. modesta* (96/97/1).

Sister to these wild species were two major cultivated clades: the *B. glabra* clade (95/100/1; containing *B. glabra*, ‘Pedro’, ‘Barbara Karst’, ‘Mrs. Eva’, ‘Mrs. Eva White’ and ‘Elizabeth Angus’) and the *B.* × *buttiana* clade (93/99/1) which comprised 16 double-bracted cultivars (‘Cherry Blossom’, ‘Los Banos Beauty’, ‘Mahara’, ‘Rose Ville Delight’, ‘Carson’s Gold’, ‘Dania White’, ‘Mrs. Eusenia Raja Singh’, ‘Mahara Pink’, ‘Mahara Ice Cream’, ‘SF Marble’, ‘Aussie Gold Splash’, ‘SF Ruby Mahara’, ‘Marietta’, ‘Mahara Variegated’, ‘Lizard Double Orange’ and ‘Cinderella’) together with three single-bracted accessions (‘Audrey Grey’, ‘Mrs. Butt’ and one public sequence). In contrast to the fully resolved interspecific backbone, internal nodes within the *B. glabra* and *B.* × *buttiana* clades collapsed into polytomies (Figure 3), which is expected given the minimal sequence divergence and the sequence-identical sets observed above (Supplementary Datas set S4 and S6). Because identical sequences cannot provide informative phylogenetic signals to resolve nested clades, plastome data plainly cannot separate these closely related cultivars. To better visualize these genealogical relationships without forcing artificial bifurcations on identical sequences, we constructed a plastid haplotype network (Supplementary Figure S12). The 29 cultivars resolved into five distinct haplotypes, with 15 of the 16 double-bracted cultivars sharing the predominant Hap_1 and ‘Mahara’ differing by a single mutational step (Hap_2; Supplementary Figure S12). This network intuitively confirms the tree topology, demonstrating that double-bracted cultivars universally share a virtually invariant *B.* × *buttiana* maternal background. Nevertheless, based on the well-resolved backbone of this phylogenetic framework, earlier ambiguous species and hybrid-group designations were successfully revised and annotated.

### 3.3. Sequence Similarity and Structural Variation of Plastid Genomes

To elucidate sequence conservation and divergence across the 29 sequenced *Bougainvillea* cultivars, we evaluated sequencing performance and quantified pairwise single nucleotide polymorphisms (SNPs) and insertions/deletions (InDels) (Figure 2b; Supplementary Figures S5 and S6). Per-base read depth profiles demonstrated consistently high and uniform coverage (mostly) along the entire plastomes of all 29 accessions, confirming high assembly completeness and accuracy for downstream variant identification (Figure 2a; Supplementary Figures S1 and S2). We found that all cultivars within the same species or hybrid group exhibited zero SNP differences, as observed across all 18 *B.* × *buttiana*, 5 *B. glabra*, 4 *B. peruviana*, and 2 *B. spectabilis* cultivars, indicating strict maternal lineage conservation (Figure 2b; Supplementary Data set S6). In contrast to the static SNPs, InDels captured minor mutations among closely related cultivars. InDel counts ranged from 1 to 14 (log_10_ values: 0.30–1.18) among the 18 *B.* × *buttiana* cultivars, and graded InDel divergence was detected within *B. peruviana* (up to 51 InDels, 1.72) (Figure 2b; Supplementary Table S6; Supplementary Figure S6). *B.* × *buttiana* and *B. glabra* exhibited close affinity, separated by only seven fixed SNPs (0.90) and 30–38 InDels (1.49–1.59). Conversely, substantial divergence separated both groups from *B. peruviana* and *B. spectabilis*, with pairwise variants exceeding 910 SNPs (2.96) and 2300 InDels (3.36–3.39). *B. peruviana* and *B. spectabilis* clustered as closely related sister taxa, differing by 379 SNPs (2.58) and 1100–1150 InDels (3.04–3.06) (Supplementary Data set S6; Supplementary Figures S5 and S6). Together, these results demonstrate that plastomic SNPs serve as reliable, diagnostic barcodes for distinguishing *Bougainvillea* species/hybrid origins, while InDels provide complementary resolution for differentiating cultivars within shared maternal lineages.

**Figure 4:**
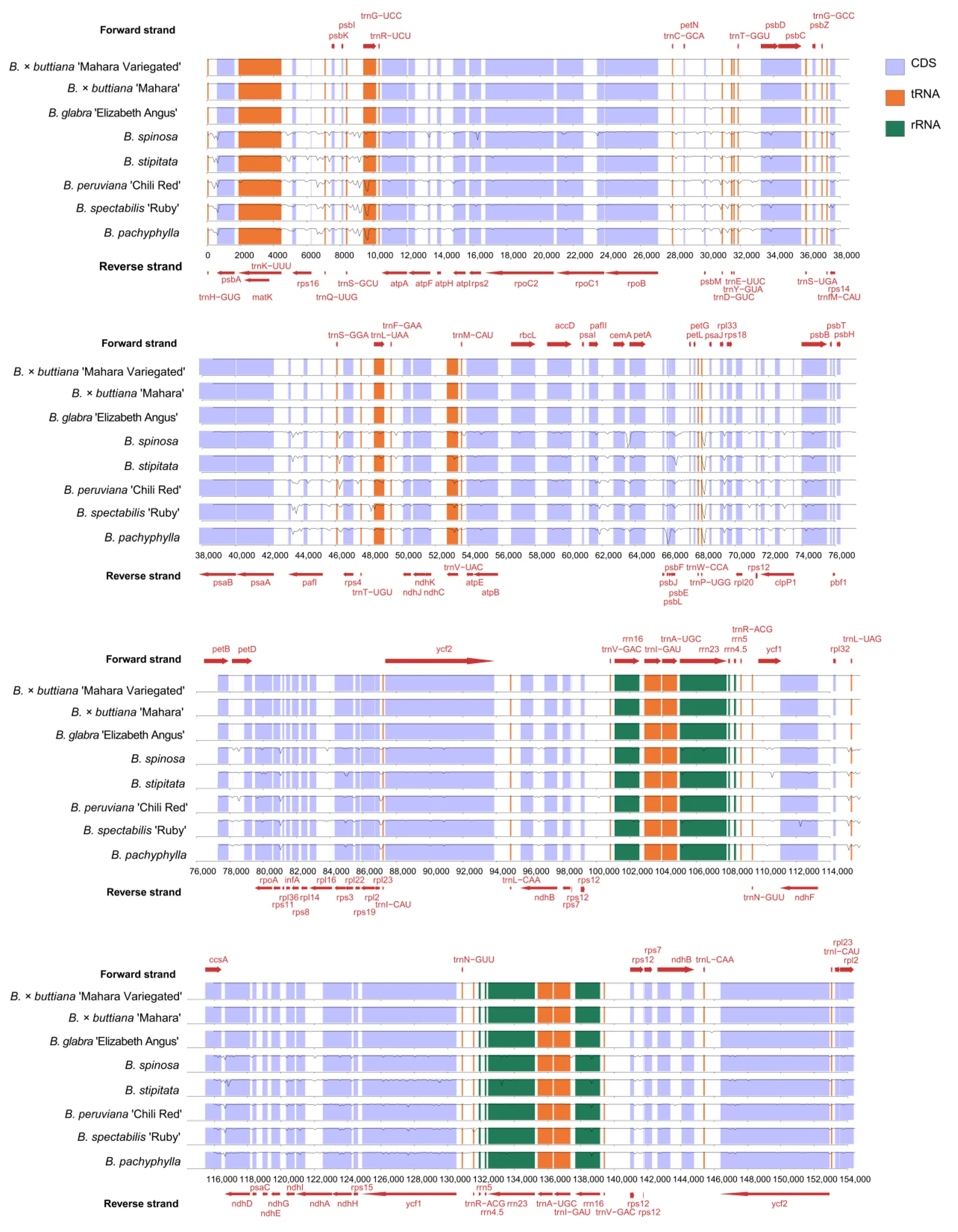
Global sequence identity alignment of eight representative *Bougainvillea* plastomes using ‘Mahara’ as a reference (mVISTA). The horizontal axis indicates the genomic coordinates (in base pairs) along the complete plastome. The vertical scale within each track represents sequence identity ranging from 50% to 100%. Red arrows denote the positions, names, and transcriptional orientations of genes on the forward and reverse strands. Functional genomic features are color-coded: protein-coding sequences (CDS) in lavender, tRNAs in orange, and rRNAs in dark green. White/unshaded areas represent non-coding regions, including introns and intergenic spacers (IGS).

**Figure 5:**
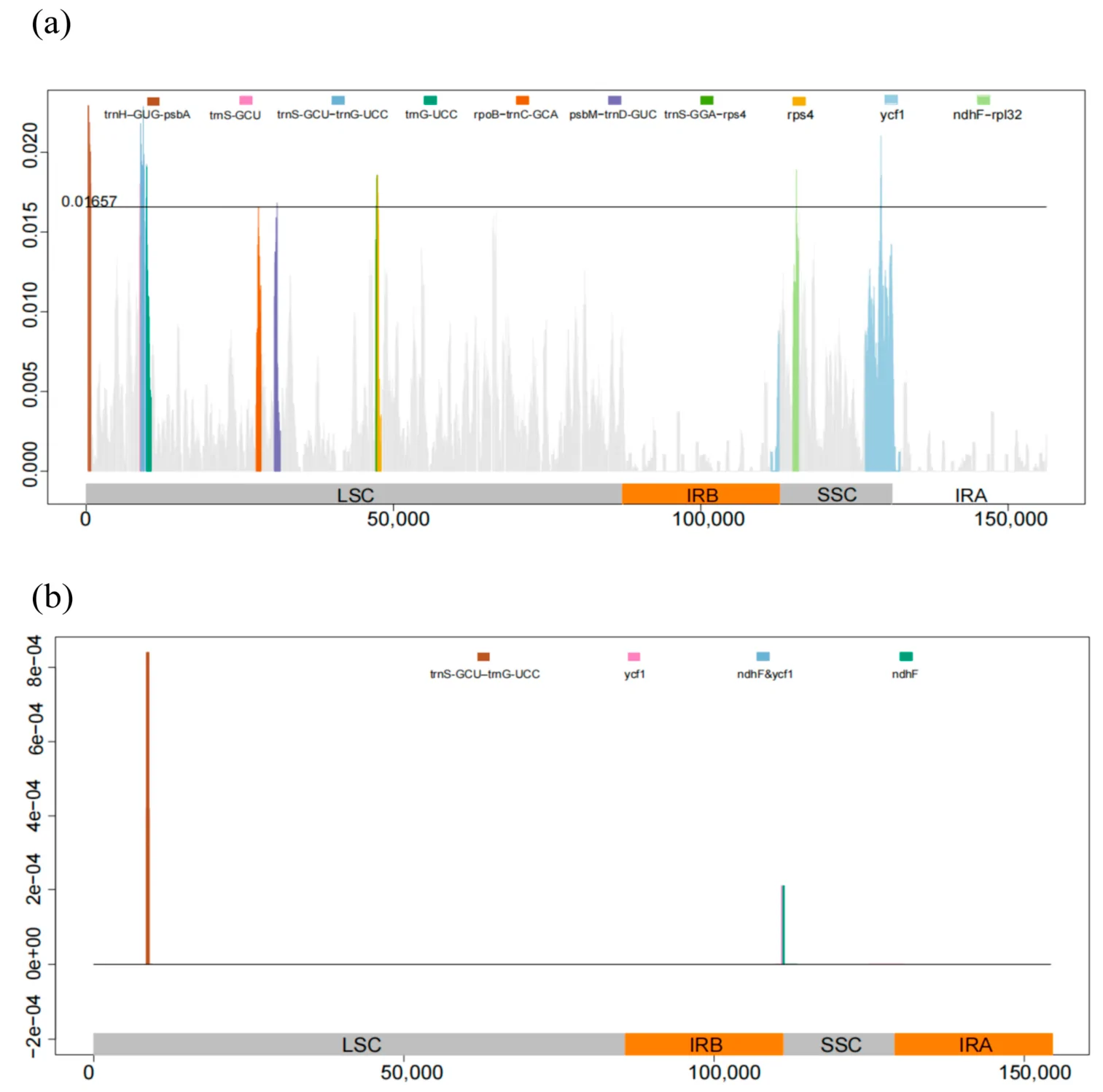
Sliding-window analysis of nucleotide diversity (Pi) across *Bougainvillea* plastomes. (**a**) Nucleotide diversity (Pi) across 17 representative *Bougainvillea* accessions capturing interspecific diversity. The horizontal line denotes the empirical threshold of Pi = 0.01657 (corresponding to the value of the 10th-highest peak), defining the top ten most variable divergence hotspots (labeled with color-coded boxes). (**b**) Nucleotide diversity (Pi) recalculated exclusively within the *B.* × *buttiana* clade (all 18 *B.* × *buttiana* cultivars), illustrating the extreme sequence conservation (Pi values dropping near zero, ranging from 0 to 8.4 × 10^−4^), with only four variable regions (*trnS-GCU–trnG-UCC*, *ycf1*, *ndhF–ycf1*, and *ndhF*) exhibiting detectable polymorphic peaks (indicated by colored boxes). In both panels, the *x*-axis represents the genomic coordinates, the *y*-axis shows Pi values, and the bottom horizontal bars depict the quadripartite plastome organization (LSC, IRB, SSC, and IRA).

To determine the genomic distribution of these variable sites across the plastome, 17 representative plastomes were selected based on the phylogenetic framework and subjected to comparative analysis using MAFFT. Spanning all major clades, these accessions comprised 1–3 representatives from each lineage alongside seven cultivars from the *B.* × *buttiana* clade, specifically incorporating cultivars that share identical sequences despite displaying distinct phenotypic traits, to scrutinize fine-scale intra-clade variation and conservation. A whole-plastome alignment (Supplementary Figure S13) revealed higher sequence divergence in the LSC and SSC regions than in the IRs, and more in non-coding than in coding sequence, with the intergenic spacers (IGS) the most variable of all. mVISTA, run against ‘Mahara’ (Figure 4), put the divergence hotspots mostly in IGS—*trnH-GUG*–*psbA*, *rps16–trnQ-UUG*, *psbI–trnS-GCU*, *trnS-GCU–trnG-UCC*, *rpoB–trnC-GCA*, *psaA–pafI*, *pafI–trnS-GGA*, *ndhC–trnV-UAC*, *accD–psaI*, *petA–psbJ*, and *psbE–petL*—with further divergence in the intron-bearing *pafI*, *trnL-UAA*, and *trnI-GAU*. Across all 17 accessions, sliding-window scan of nucleotide diversity (Pi) gave values from 0 to 0.02293. Ranking the sliding windows by nucleotide diversity identified the ten most variable divergence hotspots across the plastome, corresponding to an empirical cut-off of Pi ≥ 0.01657: *trnH-GUG–psbA*, *trnS-GCU*, *trnS-GCU–trnG-UCC*, *trnG-UCC*, *rpoB–trnC-GCA*, *psbM–trnD-GUC*, *trnS-GGA–rps4*, *rps4*, *ycf1*, and *ndhF–rpl32*. The two most variable, *trnH-GUG–psbA* and *trnS-GCU–trnG-UCC*, peaked at Pi = 0.02293 and 0.02288 (Figure 5a; Supplementary Datas set S7 and S8). However, when nucleotide diversity was recalculated exclusively within the *B.* × *buttiana* clade, the plastome was found to be exceptionally conserved, with Pi values dropping near zero across the entire genome (ranging from 0 to 0.00084, Figure 5b; Supplementary Datas set S7 and S8). In particular, the interspecific hotspots failed to exhibit polymorphism among the double-bracted cultivars. This confirms that while these high-Pi regions serve as effective DNA barcodes for discriminating *Bougainvillea* species and major lineages, plastid markers lack sufficient resolution to distinguish most cultivars within the *B.* × *buttiana* complex, reflecting their very recent shared maternal origin and clonal propagation history.

### 3.4. Comparison of IR/SC Boundaries

**Figure 6:**
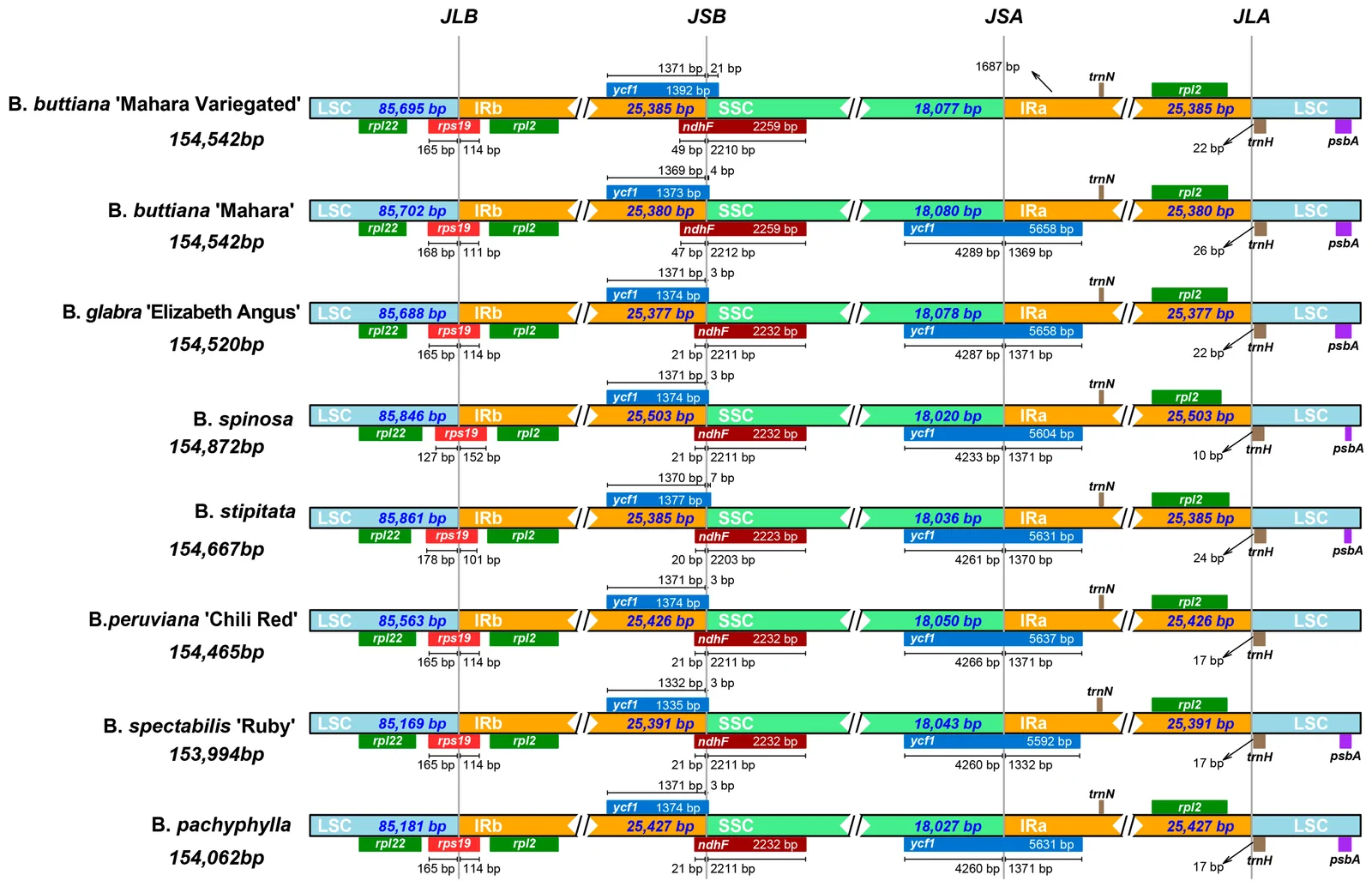
Comparison of the borders between large single-copy (LSC), small single-copy (SSC), and inverted repeat (IRa/IRb) regions among eight representative *Bougainvillea* plastomes. Vertical gray lines indicate the four quadripartite junctions: JLB (LSC/IRb), JSB (IRb/SSC), JSA (SSC/IRa), and JLA (IRa/LSC). Genes flanking or straddling the boundaries are represented as colored boxes above or below the genomic tracks. Numbers displayed within the colored genomic blocks indicate the lengths of the corresponding LSC, IRb, SSC, and IRa regions (in base pairs, bp). Total plastome sizes are shown on the left below each taxon name. Numbers positioned along the boundaries indicate the exact distance (in bp) of gene fragments extending across junctions or the distance between genes (such as trnH) and adjacent boundaries.

We then compared the four quadripartite junctions across the 17 plastomes (Figure 6; Supplementary Figure S14; Supplementary Data set S9). Gene content and order were much the same throughout, but every junction shifted somewhat as the IRs expanded and contracted. At the LSC/IRb junction (JLB) *rps19* usually straddled the boundary, reaching 165 bp into the LSC and 114 bp into the IRb, but the split moved to 127/152 bp in *B. spinosa*, 178/101 bp in *B. stipitata*, and 168/111 bp in ‘Mahara’. At the IRb/SSC junction (JSB) *ndhF* was 2232 bp (21 bp in the IRb) in most plastomes and 2223 bp (20 bp in the IRb) in *B. stipitata*, but reached 2259 bp in ‘Mahara Variegated’ and ‘Mahara’ (49 bp and 47 bp in the IRb, respectively); an *ndhF*–*ycf1* overlap was present here in every genome. At the SSC/IRa junction (JSA), the boundary was located within the *ycf1* gene, extending 1332 bp into the IRa region in *B. spectabilis* and ‘Ruby’ 1369 bp in ‘Mahara’, 1370 bp in *B. stipitata*, and 1371 bp in the remaining accessions. Due to the inverted repeat, this resulted in a corresponding partially duplicated pseudogene (*ycf1*) of identical length located in the IRb region at the IRb/SSC junction. Notably, ‘Mahara Variegated’ exhibited a particularly distinct pattern, in which the complete *ycf1* copy spanning the SSC/IRa junction was pseudogenized, whereas the IRb-derived duplicated fragment represented a partial copy of *ycf1* (Supplementary Figure S7). At the IRa/LSC junction (JLA), within the *rpl2*–*trnH* spacer, the gap between *trnH* and the boundary changed only slightly: 10 bp in *B. spinosa*; 17 bp in *B. pachyphylla*, *B. spectabilis*, ‘Ruby’, ‘Chili Purple’, ‘Chili Red’, and *B. peruviana*; 22 bp in *B. glabra*, ‘Elizabeth Angus’, ‘Mahara Variegated’, ‘Marietta’, ‘Dania White’, *B.* × *buttiana*, ‘SF Ruby Mahara’, and ‘Los Banos Beauty’; 24 bp in *B. stipitata*; and 26 bp in ‘Mahara’.

## 4. Discussion

### 4.1. Plastome Structural Conservation, Variation, and Marker Utility in Bougainvillea

The 29 plastomes are highly conserved, and all maintain the quadripartite plan typical of flowering plants, as reported previously for the genus. [12,13]. Sixteen share an identical length of 154,542 bp, and GC content stays within a narrow band both overall (36.5–36.6%) and region by region. A plastome conserved to this degree through long-term cultivation and selection suggests that the basic architecture and base composition of the *Bougainvillea* plastome are under strong constraint. That several groups of cultivars—the ‘Flame’–’Ruby’ pair and the 13-cultivar *B.* × *buttiana* set—are completely sequence-identical reflects shared maternal lineages, but also highlights the inherent resolution limits of plastid genomes at the cultivar level. In horticultural woody taxa like *Bougainvillea*, distinct cultivars often arise from recent somatic bud mutations (bud sports) that alter bract color or morphology, or from hybridization events sharing the same maternal lineage but different pollen donors. Because plastid genomes evolve at a relatively slow substitution rate and follow uniparental maternal inheritance, they remain invariant across such rapid phenotypic diversifications and cannot capture paternal contributions over short breeding timescales. Indeed, for cultivars exhibiting distinct bract colors (e.g., red, pink, white, and orange forms) or leaf variegations display marked morphological differentiation, their identical plastomes clearly reflect recurrent somatic bud mutations (bud sports). Therefore, while plastomes reliably establish maternal lineages, resolving cultivars with identical plastomes will ultimately require high-resolution nuclear genomic markers.

Despite the overall conservation of the plastomes, there is still modest structural variation. The cultivated plastomes are a little shorter than those of wild species such as *B. spinosa* and *B. stipitata*; rather than reading this as simple lineage retention, we think it reflects local rearrangement—IR expansion and contraction and the movement of genes near the IR/SC junctions [44,45,46,47]. Gene number was 130–131. ‘Mahara Variegated’ contained one fewer annotated functional gene than most previously reported *Bougainvillea* plastomes. This difference was likely associated with pseudogenization of the complete *ycf1* copy spanning the SSC/IRa junction rather than loss of the entire *ycf1* locus. The IR/SC junctions are highly dynamic regions in plastid genomes, and IR expansion or contraction can modify the structure of boundary-associated genes, leading to partial duplication, truncation, premature termination, or pseudogenization of *ycf1*-related copies [46,47]. Given the high evolutionary variability of *ycf1* and its frequent localization at the SSC/IRa boundary [46,47], the pattern observed in ‘Mahara Variegated’ represents a definitive, fixed structural mutation rather than an assembly artifact. We identified 18 duplicated genes in the IRs, in agreement with earlier reports, demonstrating that the overall gene complement in the IR regions remains highly conserved across cultivars despite slight fluctuations at the IR boundaries.

Overall, the *Bougainvillea* plastome displays strong structural conservation punctuated by localized hotspots of variation—namely IR-boundary shifts, lineage-specific *ycf1* pseudogenization, and variable intergenic regions. Although variegated cultivars are often suspected to harbor plastome heteroplasmy or somatic chimerism, the exceptionally high allele frequency (99.85% at 2045× depth) and cloning confirmation of the truncated *ycf1* in ‘Mahara Variegated’ demonstrate that this mutation has become completely homoplasmic. Notably, among the variegated cultivars examined in this study, this *ycf1* pseudogenization was exclusively detected in ‘Mahara Variegated’, whereas other variegated cultivars (e.g., ‘Mrs. Eusenia Raja Singh’ and ‘Lizard Double Orange’) retained intact *ycf1* coding sequences. This indicates that *ycf1* pseudogenization is unlikely to be the universal causal driver of leaf variegation in *Bougainvillea*—which may instead be governed by nuclear genes or other regulatory pathways—but rather represents a stable, lineage-specific mutation that serves as an unambiguous molecular hallmark for ‘Mahara Variegated’.

Non-coding sequence, under weaker constraint than coding sequence, tends to change faster, and IGS in particular has long been the go-to source for species identification, phylogenetics, and marker work [48,49,50,51]. Universal markers such as *trnL–trnF*, *trnH–psbA*, and *psbK–psbI* have discriminated angiosperms, ferns, and bryophytes since the 1990s [48,50,51]. Finding the fast-changing parts of the *Bougainvillea* plastome is therefore the first step toward markers that can separate its cultivars and support germplasm work. Here the variation was not spread evenly but banked up in a few regions. Comparative alignments (MAFFT and mVISTA) and the sliding-window scan agreed in placing these in the LSC and SSC, mostly in IGS, and two loci stood out—*trnH-GUG*–*psbA* and *trnS-GCU–trnG-UCC*, at Pi = 0.02293 and 0.02288—as prominent DNA barcodes for species-level discrimination (Figure 5a). However, when evaluated strictly within the *B.* × *buttiana* complex, nucleotide diversity dropped near zero across the entire genome (Pi ≤ 8.4 × 10^−4^; Figure 5b), demonstrating that these interspecific hotspots lack sufficient polymorphism to separate closely related double-bracted cultivars. Lin et al. [14] had already shown that SNPs in *trnS-GCU–trnG-UCC* distinguish *B.* × *buttiana* cultivars, so our result and theirs reinforce each other. Collectively, these hotspots provide informative markers for species-level delimitation, identifying wild relatives, and tracing major maternal lineages, though they provide limited resolution for discriminating closely related cultivars.

### 4.2. Phylogenetic Placement and Taxonomic Reappraisal of Bougainvillea Cultivars

The plastome phylogenomic tree robustly resolves the maternal relationships of the cultivars. Lineages tied to *B. pachyphylla*, *B. spectabilis*, and *B. peruviana* diverged deep within the topology. Cultivars ‘Ruby’ and ‘Flame’ grouped with *B. spectabilis*, confirming they possess a *B. spectabilis*-type plastome, consistent with the findings of Wu et al. [17]. Cultivars ‘Chili Purple’, ‘Chili Orange’, ‘Chili Red’, and ‘Chili Yellow’ formed a strongly supported clade with B. peruviana, providing novel whole-plastome evidence for the maternal placement of this series. Furthermore, ‘Pedro’, ‘Barbara Karst’, ‘Mrs. Eva’, ‘Mrs. Eva White’, and ‘Elizabeth Angus’ clustered within the *B. glabra* lineage; while the affinity of ‘Elizabeth Angus’ was previously known, maternal origins for the other four are firmly established here, offering molecular support for classifying *B. glabra*-derived cultivars. Crucially, all 16 double-bracted cultivars investigated here were placed within the *B.* × *buttiana* plastid clade. The newly sequenced accessions—‘Marietta’, ‘Dania White’, ‘Mahara Variegated’, ‘SF Ruby Mahara’, and ‘Mahara’—clustered closely with previously reported double-bracted cultivars (‘Los Banos Beauty’, ‘Carson’s Gold’ [syn. ‘Double Yellow’], and ‘Rose Ville Delight’ [syn. ‘Double Salmon’]) [17], as well as single-bracted *B.* × *buttiana* accessions such as ‘Audrey Grey’ and ‘Mrs. Butt’. Given the maternal inheritance of angiosperm plastomes and the widespread presence of sequence-identical accessions, these findings point to a shared, or very nearly common, *B.* × *buttiana* maternal ancestry for double-bracted cultivars. This shared maternal background clarifies a historical taxonomic discrepancy: whereas Sindhu et al. [10] allied ‘Los Banos Beauty’ with *B. glabra*, our whole-plastome tree decisively places its maternal lineage within *B.* × *buttiana*, in line with the nomenclature proposed by Wu et al. [17].

In previous studies, double-bracted cultivars often appeared scattered across phylogenetic trees, likely due to restricted sampling, under-representation of double-bracted forms, or limited resolution of single-gene markers [17]. Through comprehensive sampling and complete plastome sequences, we demonstrate that despite their diverse bract colors and morphologies, double-bracted forms universally share a *B.* × *buttiana* maternal background. This finding aligns with their biology: lacking functional tubular flowers, they are propagated strictly through vegetative cuttings and remain genetically stable over horticultural transfers, making their maternal plastomes faithful tracers of their initial origin. However, plastid markers alone cannot untangle reticulate hybrid ancestry or capture nuclear genomic dynamics. Consequently, taxonomic revisions based solely on maternal markers must be interpreted with caution. Evolutionary processes such as chloroplast capture via introgressive hybridization, incomplete lineage sorting (ILS), and asymmetric cytonuclear discordance can cause plastid gene trees to deviate from the true species phylogeny. Therefore, the taxonomic placements proposed here should be viewed as reliable hypotheses of maternal affinity rather than definitive organismal circumscriptions. To rigorously validate these maternal assignments and reconstruct complete organismal genealogies, future studies should employ integrative approaches: (1) High-throughput nuclear phylogenomics (e.g., Hyb-Seq targeting Angiosperms353 or whole-genome resequencing) to detect bi-parental allelic contributions, quantify nuclear admixture, and resolve reticulate networks; (2) Cytogenetic and ploidy assessments, including flow cytometry and genomic in situ hybridization (GISH), to determine polyploidization and identify parental subgenomes; and (3) Integrative taxonomy combining nuclear datasets with micromorphological characters (e.g., bract and perianth trichome architecture) and horticultural breeding pedigrees. Such multidimensional evidence will be indispensable for establishing an authoritative taxonomic framework for *Bougainvillea*.

### 4.3. Implications for Bougainvillea Breeding and Germplasm Utilization

Beyond evolutionary insights, our findings offer practical guidance for *Bougainvillea* breeding and germplasm utilization. First, because double-bracted cultivars are completely sterile and cannot be crossed directly, confirming their origin as recurrent somatic bud sports within *B.* × *buttiana* provides a dual-faceted breeding roadmap. On the one hand, novel double-bracted forms within this lineage are best achieved through targeted mutagenesis or systematic bud-sport selection rather than futile trial-and-error hybridization. On the other hand, the phylogenomic restriction of all currently known double-bracted cultivars to *B.* × *buttiana* reveals a major untapped opportunity: inducing somatic mutations in other lineages—most notably *B. glabra* and *B. spectabilis*—could break existing phenotypic bottlenecks and yield unprecedented double-bracted cultivars displaying novel color spectra, such as vibrant purple or magenta. Second, for fertile single-bracted cultivars, clarifying maternal backgrounds helps breeders avoid redundant crosses between cultivars that, despite differing in bract color, share the same clonal maternal foundation. Finally, the extremely low plastome divergence observed across commercial cultivars underscores their narrow maternal genetic base, highlighting the urgent necessity of introgressing wild germplasm (such as *B. spinosa*) via fertile bridge lines to broaden genetic diversity and enhance stress resilience in future breeding programs.

## 5. Conclusions

In conclusion, this study establishes a comprehensive plastid phylogenomic framework that elucidates the maternal origins and evolutionary history of cultivated *Bougainvillea*, with a specific focus on double-bracted forms. Our comparative genomic analyses demonstrate that double-bracted phenotypes predominantly derived from the *B.* × *buttiana* maternal lineage through recurrent somatic bud mutations rather than multiple independent hybridization events. While the high sequence conservation underscores the inherent resolution limits of maternal plastid markers for fine-scale cultivar delimitation, specific genomic features—particularly the frameshift-induced *ycf1* pseudogene in ‘Mahara Variegated’ and the identified divergent intergenic spacers—provide promising diagnostic markers for germplasm screening. Overall, these findings resolve long-standing ambiguities regarding the maternal pedigrees of cultivated double-bracted Bougainvillea, enrich valuable genomic resources, and highlight the imperative of integrating nuclear genomic approaches for future cultivar discrimination, germplasm conservation, and molecular breeding in this ornamental genus.

## Figures and Tables

**Table 1:** Gene composition of 29 newly sequenced *Bougainvillea* plastid genomes.

Category of Genes	Group of Genes	Genes
Photosynthesis	Subunits of photosystem I	*psaA*, *psaB*, *psaC*, *psaI*, *psaJ*
	Subunits of photosystem II	*psbA*, *psbB*, *psbC, psbD*, *psbE*, *psbF*, *psbJ*, *psbK*, *psbL*, *psbM*, *psbT*, *psbZ*, *psbH*, *psbI*
	Subunits of cytochrome b/f complex	*petA*, *petB*^a^, *petD*^a^, *petG*, *petL*, *petN*
	Subunits of ATP synthase	*atpA*, *atpB*, *atpE*, *atpF*^a^, *atpH*, *atpI*
	Photosystem biogenesis factor	*pbf1*
	Photosystem I assembly factors	*pafI*^b^, *pafII*
	Subunits of NADH-dehydrogenase	*ndhA*^a^, *ndhB* (×2) ^a^, *ndhC*, *ndhD*, *ndhE*, *ndhF*, *ndhG*, *ndhH*, *ndhI*, *ndhJ*, *ndhK*
	Subunits of rubisco	*rbcL*
Self-replication	RNA polymerase	*rpoA*, *rpoB*, *rpoC1*^a^, *rpoC2*
	Large ribosomal subunits	*rpl2* (×2), *rpl14*, *rpl16*^a^, *rpl20*, *rpl22*, *rpl23* (×2), *rpl32*, *rpl33*, *rpl36*
	Small ribosomal subunits	*rps2*, *rps3*, *rps4*, *rps7* (×2), *rps8*, *rps11*, *rps12* (×2)^b^, *rps14*, *rps15*, *rps16*^a^, *rps18*, *rps19*
	Ribosomal RNAs	*rrn4.5* (×2), *rrn5* (×2), *rrn16* (×2), *rrn23* (×2)
	Transfer RNAs	*trnA-UGC* (×2)^a^, *trnC-GCA*, *trnD-GUC*, *trnE-UUC*, *trnF-GAA*, *trnfM-CAU*, *trnG-GCC*, *trnG-UCC*^a^, *trnH-GUG*, *trnI-CAU*(×2), *trnI-GAU* (×2)^a^, *trnK-UUU^a^*, *trnL-CAA* (×2), *trnL-UAA^a^*, *trnL-UAG*, *trnM-CAU*, *trnN-GUU* (×2), *trnP-UGG*, *trnQ-UUG*, *trnS-GCU*, *trnR-ACG* (×2), *trnR-UCU*, *trnS-GGA*, *trnS-UGA*, *trnT-GGU*, *trnT-UGU*, *trnV-GAC* (×2), *trnV-UAC^a^*, *trnW-CCA*, *trnY-GUA*
Other genes	Subunit of acetyl-coA-carboxylase	*accD*
	c-type cytochrome synthesis gene	*ccsA*
	Envelop membrane protein	*cemA*
	Protease	*clpP1* ^b^
	Translational initiation factor	*infA*
	Maturase	*matK*
Unknown function	Conserved open reading frames	*ycf1* (×2), *ycf2* (×2)

Notes: (×2) indicates genes duplicated in the inverted repeat (IR) regions; “^a^” indicates genes containing one intron; and “^b^” indicates genes containing two introns.

## Data Availability

The complete plastid genome sequences generated in this study have been deposited in NCBI GenBank under the accession numbers PZ569225-PZ569253 (listed in Supplementary Data set S1). All other data supporting the findings of this study are included in this article and its Supplementary Information files.

## Supplementary Materials

The following supporting information can be downloaded at: https://www.mdpi.com/article/10.3390/biology15201873/s1, Figure S1: Plastome sequencing-depth profiles of 29 *Bougainvillea* accessions; Figure S2: Mean per-base sequencing quality across the plastomes of 29 *Bougainvillea* accessions; Figure S3: Circular map of the *B*. × *buttiana* ‘Mahara’ plastome; Figure S4: Collinearity and gene-order comparison of the complete plastomes of 29 *Bougainvillea* accessions; Figure S5: Pairwise single-nucleotide polymorphism differences among 29 *Bougainvillea* accessions; Figure S6: Pairwise insertion–deletion differences among 29 *Bougainvillea* accessions; Figure S7: Read-depth and sequence-based validation of the putative *ycf1* pseudogene in *B.* × *buttiana* ‘Mahara Variegated’; Figure S8: Structures of intron-containing genes in the plastome of *B*. × *buttiana* ‘Mahara’; Figure S9: Maximum-likelihood phylogeny inferred from the concatenated amino-acid sequences of plastid protein-coding genes. Figure S10: Phylogenetic relationships inferred from the trimAl-trimmed complete-plastome alignment of 44 taxa. Figure S11: Bayesian phylogeny inferred from the untrimmed concatenated amino-acid sequences of plastid protein-coding genes. Figure S12: Plastome haplotype relationships among 29 *Bougainvillea* accessions. Figure S13: Global sequence-identity comparison of 17 *Bougainvillea* plastomes with ‘Los Banos Beauty’ as reference (mVISTA); Figure S14: Comparison of the LSC/IR and SSC/IR junction regions among the 17 representative *Bougainvillea* plastomes; Dataset S1: Sample name, origin, accession number, and NCBI accession number of all samples; Dataset S2: Summary statistics of plastome sequencing coverage and base quality for 29 *Bougainvillea* accessions based on mapping quality-filtered reads to their respective final plastome assemblies; Dataset S3: Pairwise sequence identities (%) among the 29 complete plastomes of *Bougainvillea*; Dataset S4: Summary of plastid genome characteristics of 29 samples; Dataset S5: Per-site sequencing depth and allele proportions across the *ycf1* region of *B.* × *buttiana* ‘Mahara Variegated’; Dataset S6: Pairwise plastome-wide single-nucleotide polymorphism (SNP) and insertion–deletion (InDel) differences among 29 *B.* × *buttiana* ‘Mrs. Butt’ gainvillea cultivars; Dataset S7: Sliding-window estimates of nucleotide polymorphism across the complete plastome alignment of 17 *Bougainvillea* accessions; Dataset S8: Sliding-window estimates of nucleotide polymorphism across the complete plastome alignment of 19 *B.* × *buttiana* cultivars; Dataset S9: Lengths of plastome regions and genes associated with the four IR/SC junctions in 17 *Bougainvillea* accessions.
