# Supplementary material for: Comparative Plastid Phylogenomics of 29 Newly Sequenced Bougainvillea Cultivars Resolves the Phylogenetic Placement of Double-Bracted Cultivars
Source: Biology (Basel). 2026 Oct 17;15(20):1873. doi: 10.3390/biology15201873 (PMC13701442; doi:10.3390/biology15201873)
Supplement: Supplementary file 1 [file biology-15-01873-s001.zip › supplement Fig. S1-14final_20260923(final).pdf]

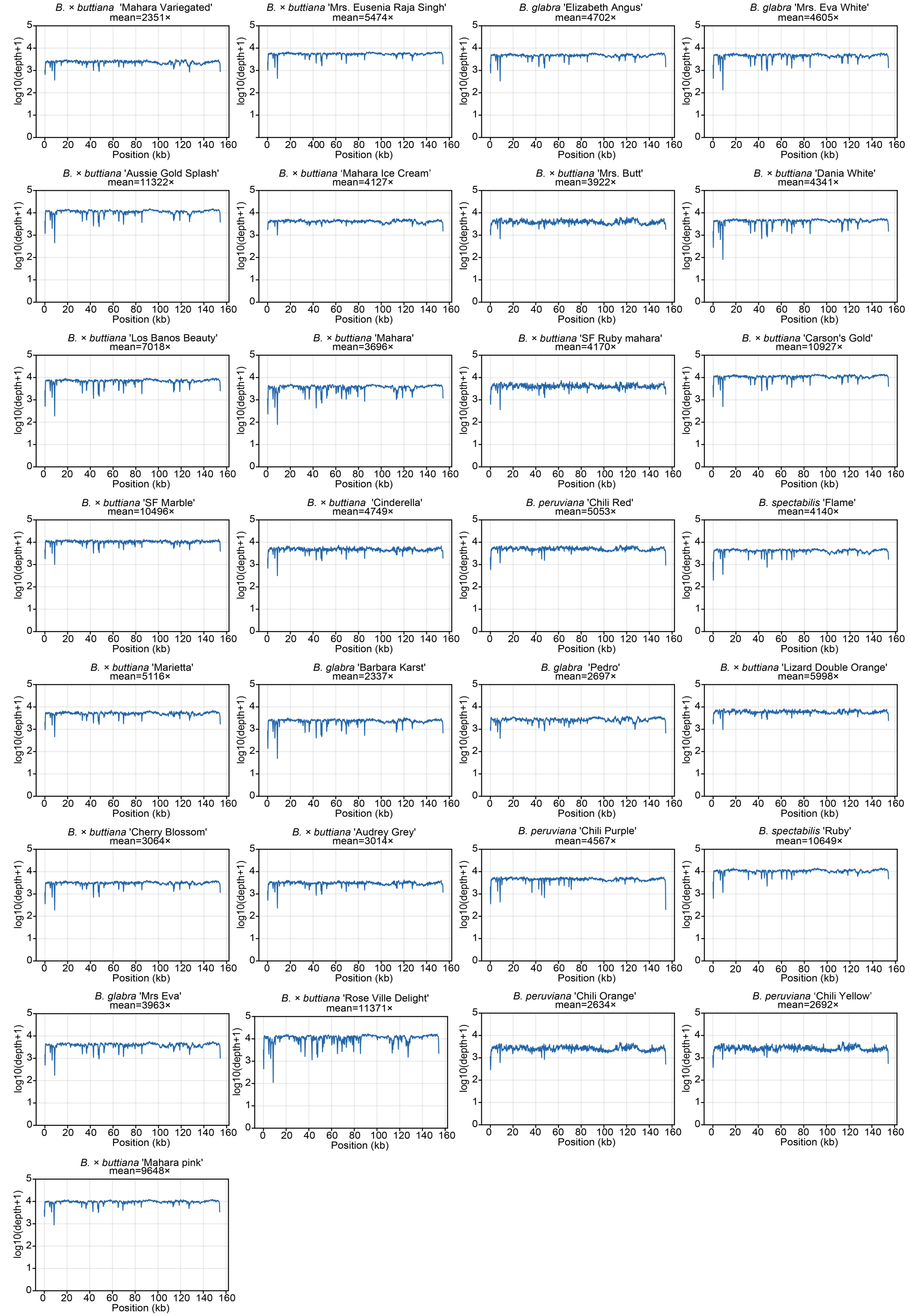

Fig. S1. Plastome sequencing-depth profiles of 29 *Bougainvillea* accessions. Quality-filtered reads were mapped back to the corresponding final plastome assemblies. For visualization, per-base sequencing depths were averaged within non-overlapping 200-bp windows and transformed as $\log_{10}$ (depth+1). Each panel represents one accession. The x-axis indicates the position within the plastome (kb), and the y-axis represents the log-transformed mean sequencing depth for each window. The value above each panel indicates the overall mean sequencing depth of the corresponding accession (×). All accessions showed high and generally continuous coverage across their complete plastomes, with only localized fluctuations in sequencing depth.

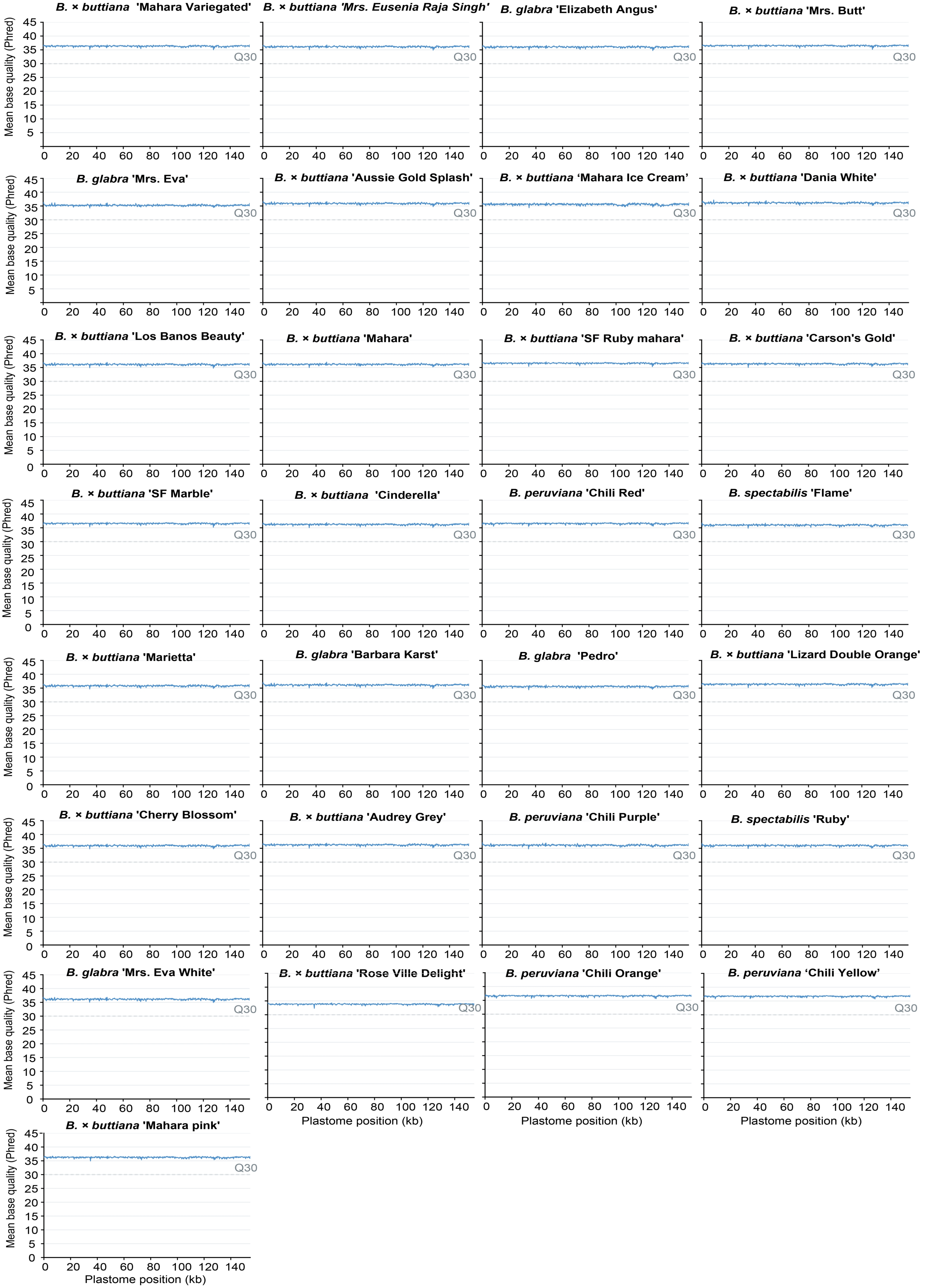

Fig. S2. Mean per-base sequencing quality across the plastomes of 29 *Bougainvillea* accessions. Quality-filtered reads were mapped back to their corresponding final plastome assemblies, and the mean base quality (Phred score) was calculated at each plastome position from the resulting alignments. Each panel represents one accession. The x-axis indicates the position along the plastome (kb), and the y-axis indicates the mean base quality. The grey dashed line represents the Q30 threshold, corresponding to an estimated base-calling error probability of 0.1% and an accuracy of 99.9%. Mean base-quality scores remained above Q30 across nearly all plastome positions in all accessions, supporting the high reliability of the plastome consensus sequences at the nucleotide level.

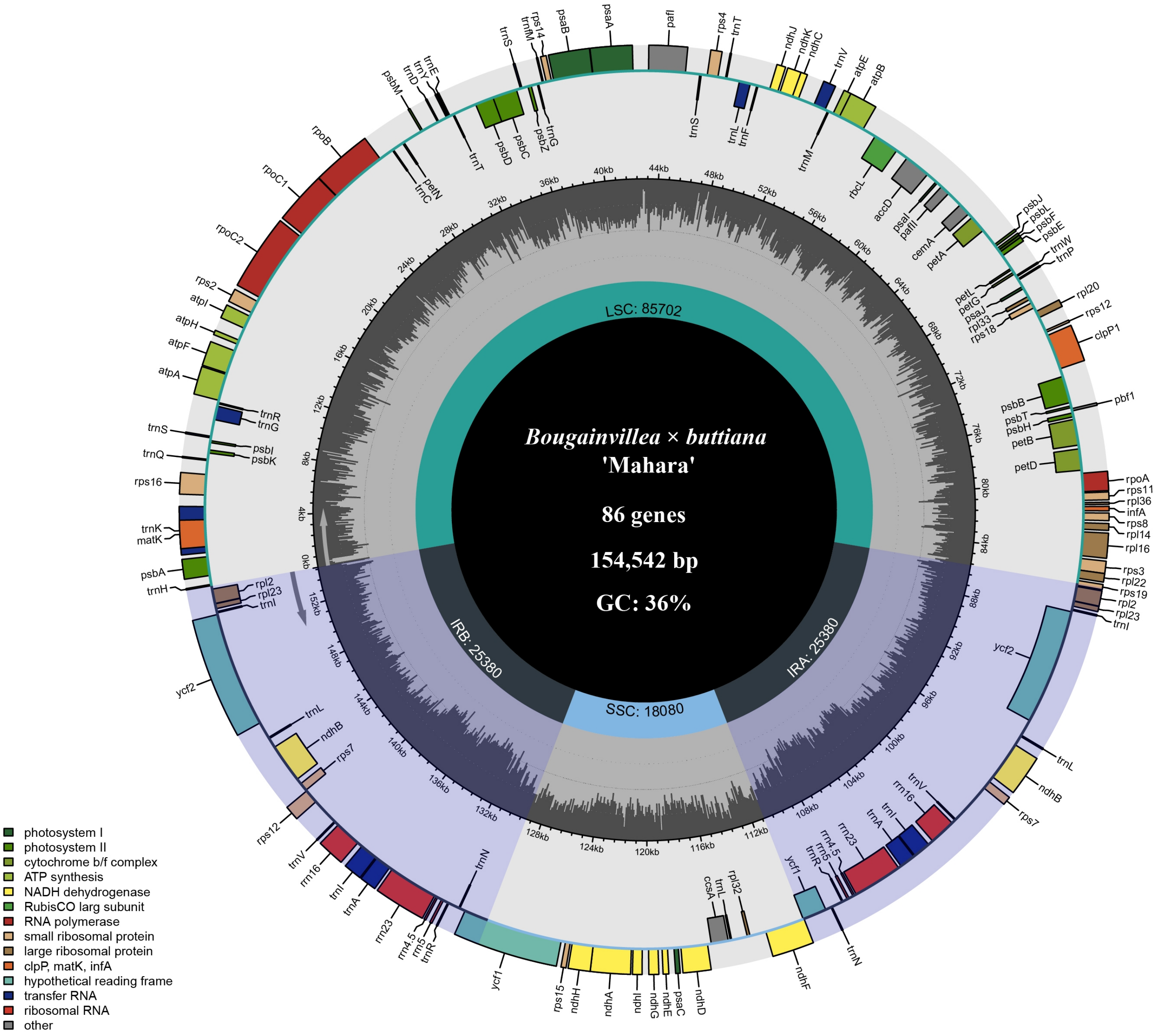

Fig. S3. Circular map of the *B.* × *buttiana* 'Mahara' plastome. The plastome is 154,542 bp in length and displays the typical quadripartite structure, comprising an 85,702-bp large single-copy (LSC) region, an 18,080-bp small single-copy (SSC) region, and two 25,380-bp inverted-repeat regions (IRa and IRb). Genes located outside and inside the outer circle are transcribed clockwise and counterclockwise, respectively. Different colors indicate genes belonging to different functional categories, as shown in the legend. The inner histogram represents variation in GC content across the plastome. The genome contains 86 unique genes and has an overall GC content of 36%.

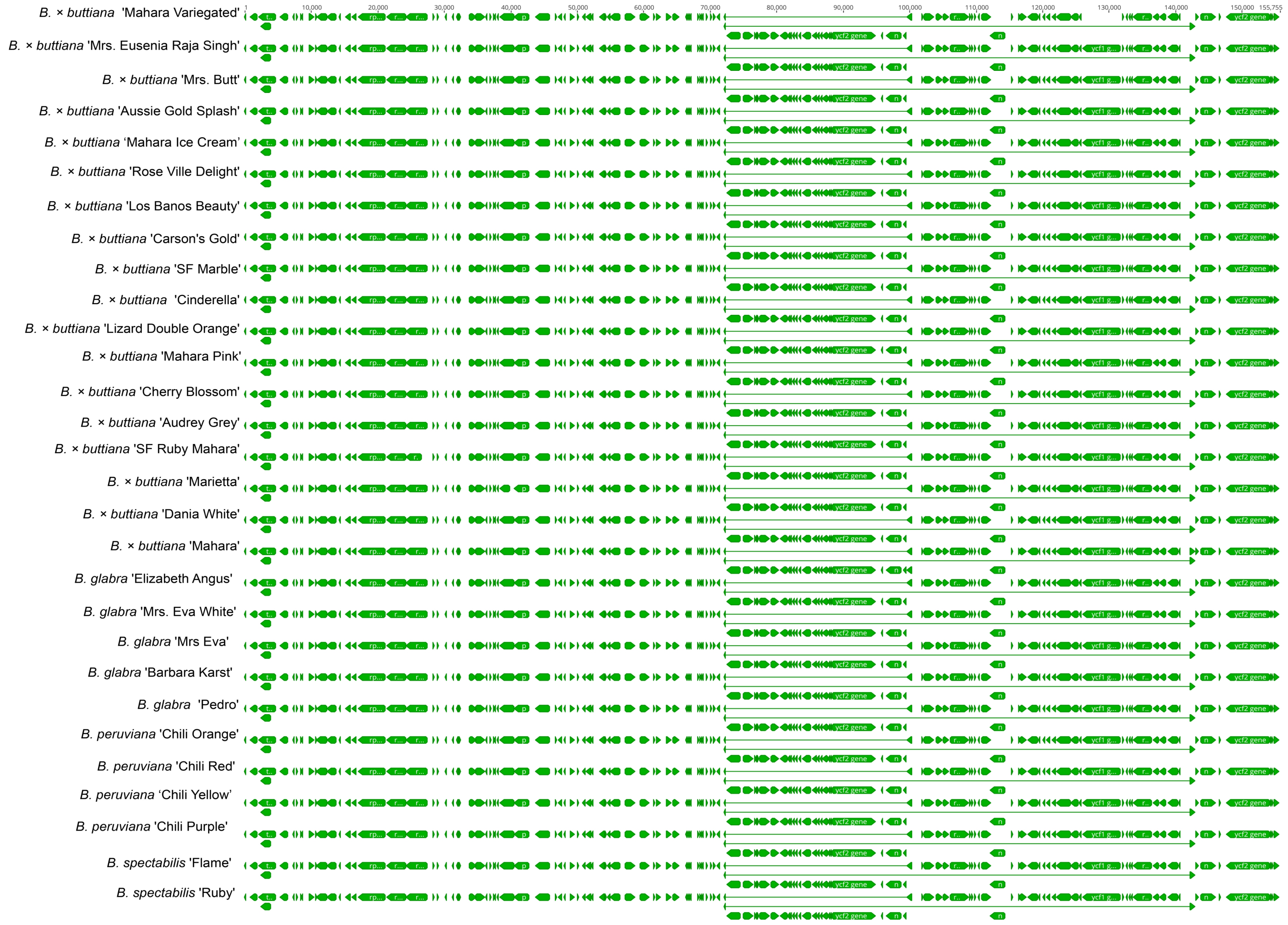

Fig. S4. Collinearity and gene-order comparison of the complete plastomes of 29 *Bougainvillea* accessions. Whole-plastome collinearity was assessed using progressiveMauve after linearizing all plastomes and arranging them in the same orientation. Green arrows represent annotated genes; their genomic positions and arrow directions indicate gene locations and transcriptional orientations, respectively. The scale at the top indicates plastome coordinates in base pairs. The 29 plastomes exhibited consistent gene order and transcriptional orientation, with no evident inversions, translocations, or other large-scale structural rearrangements, supporting a highly conserved plastome structure across the examined *Bougainvillea* accessions.

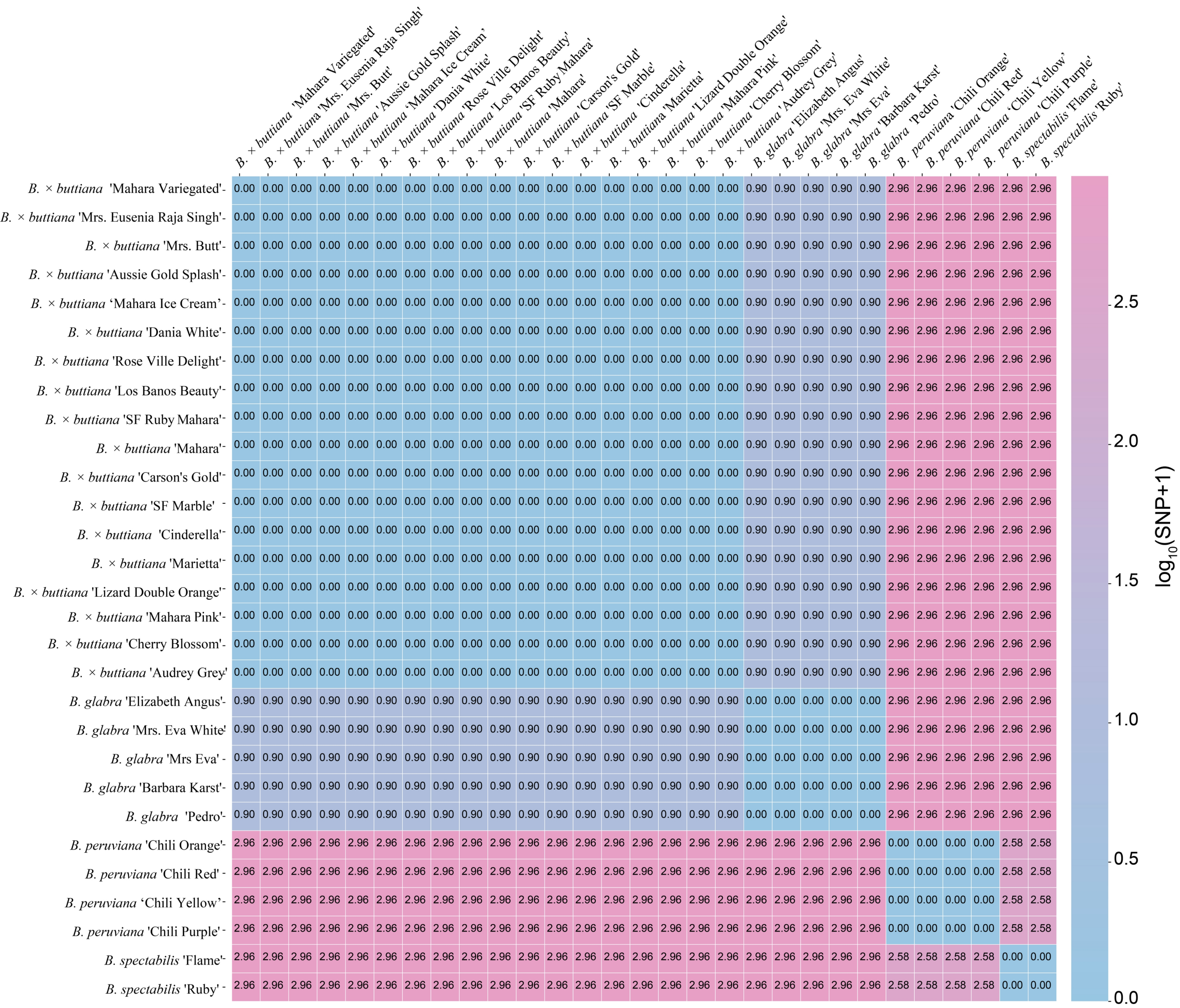

Fig. S5. Pairwise single-nucleotide polymorphism differences among 29 *Bougainvillea* accessions. Pairwise SNP differences were calculated from the whole-plastome sequence alignment and visualized after transformation as $\log_{10}$ (SNP count+1). The numerical value in each cell represents the log-transformed number of SNP differences between the corresponding pair of accessions. The color gradient from blue to pink indicates increasing pairwise SNP divergence. Diagonal cells represent self-comparisons and therefore have a value of zero. SNP divergence was generally low among accessions assigned to the same taxonomic group but substantially higher between different groups.

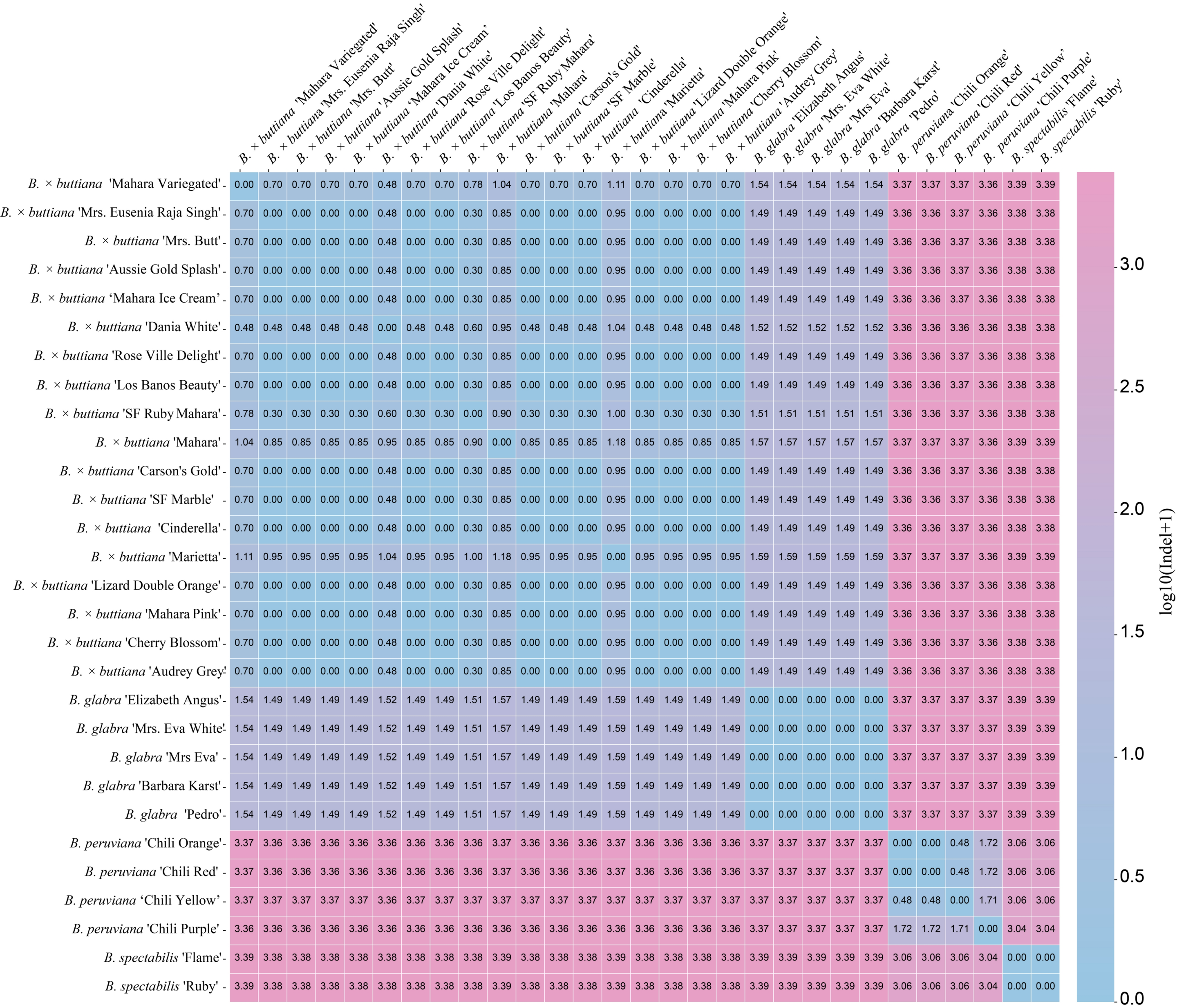

Fig. S6. Pairwise insertion–deletion differences among 29 *Bougainvillea* accessions. Pairwise insertion–deletion (InDel) differences were calculated from the whole-plastome sequence alignment and visualized after transformation as $\log_{10}$(InDel count+1). The numerical value in each cell represents the log-transformed number of InDel differences between the corresponding pair of accessions. The color gradient from blue to pink indicates increasing pairwise InDel differences. Diagonal cells represent self-comparisons and therefore have a value of zero. Pairwise InDel differences were generally limited among accessions assigned to the same hybrid or species group but were substantially greater between different taxonomic groups.

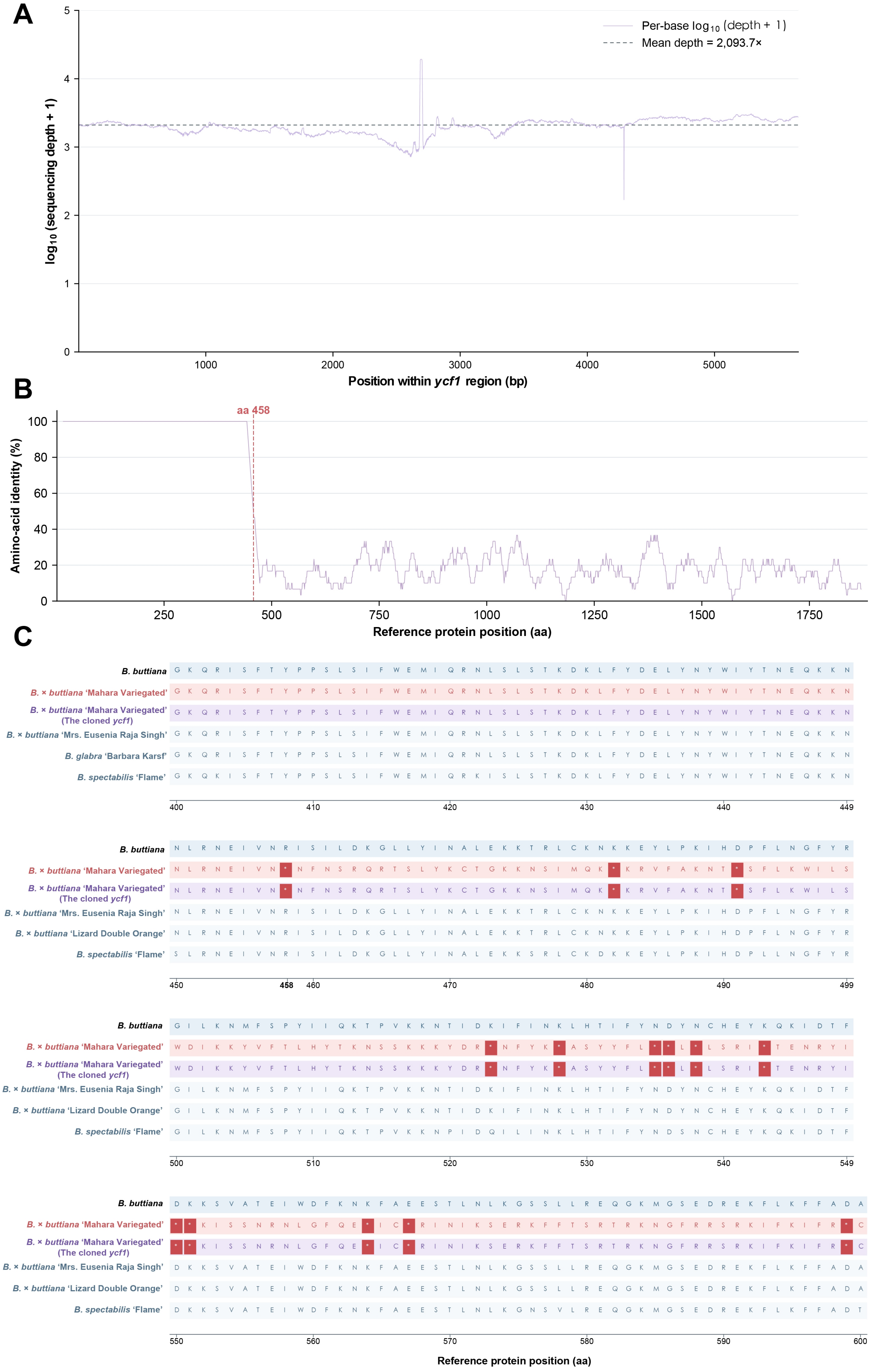

Fig. S7. Read-depth and sequence-based validation of the putative *ycf1* pseudogene in *B.* × *buttiana* 'Mahara Variegated'.
(A) Per-base sequencing depth across the complete *ycf1* region after mapping the trimmed reads back to the final plastome assembly. Depth is expressed as $\log_{10}$ (depth+1), and the dashed horizontal line indicates the mean depth of 2,093.7×. Primary alignments were retained without a mapping-quality cutoff (MAPQ ≥ 0), while bases with Phred quality scores <20 were excluded. (B) Sliding-window amino-acid identity between the predicted *ycf1* protein of 'Mahara Variegated' and the reference sequence. The red dashed line marks the putative frameshift at amino-acid position 458, after which sequence identity decreases sharply. (C) Amino-acid sequence alignment of the reference, the assembled 'Mahara Variegated' sequence, the independently cloned *ycf1* sequence, and representative *Bougainvillea* accessions across reference positions 400–600. The assembled and cloned 'Mahara Variegated' sequences show the same frameshift-associated divergence and internal stop codons downstream of position 458. Asterisks and dark-red cells indicate internal stop codons.

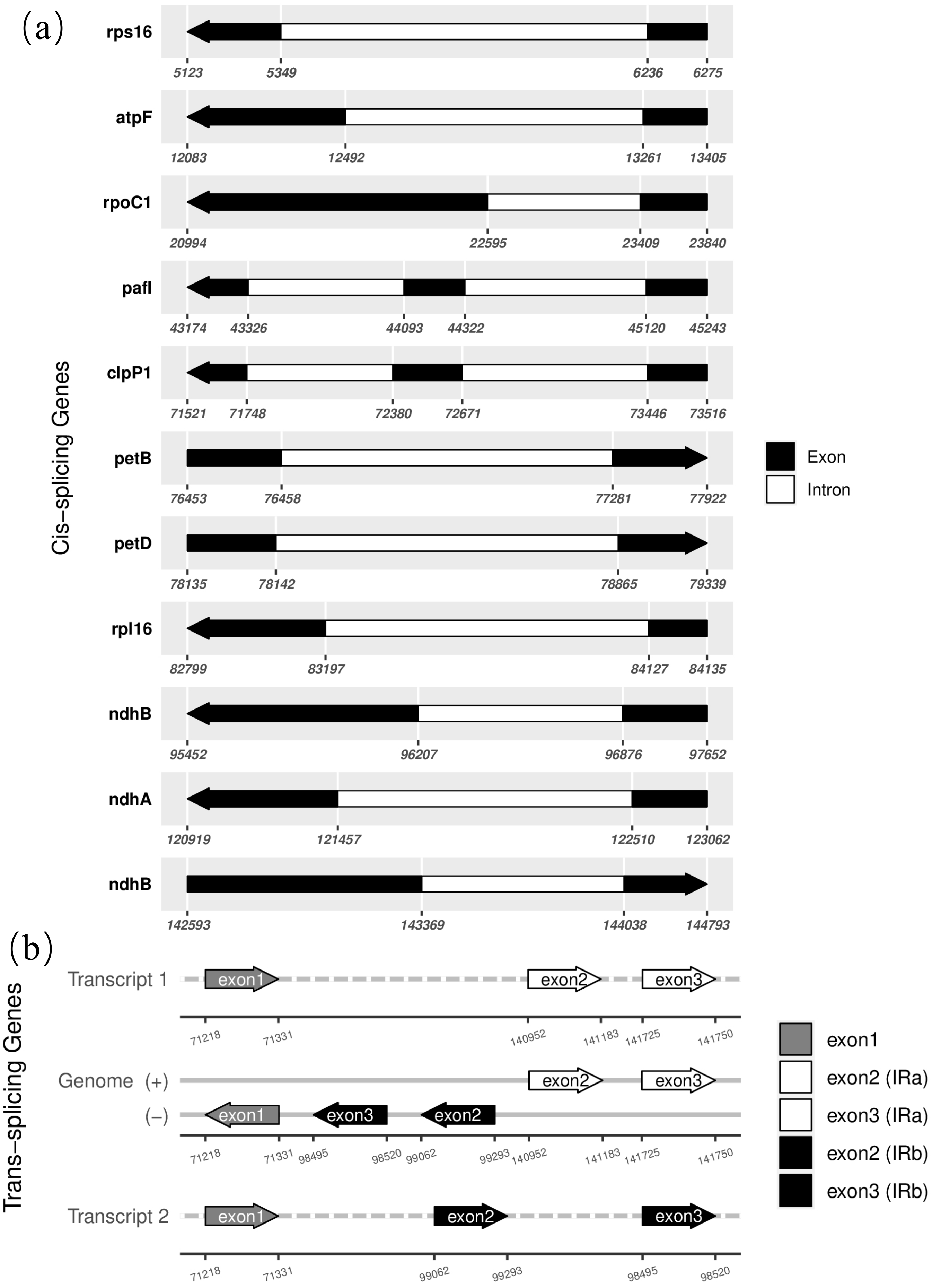

Fig. S8. Structures of intron-containing genes in the plastome of *B.* × *buttiana* 'Mahara'. (a) Structures of the cis-spliced genes *rps16*, *atpF*, *rpoC1*, *pafI*, *clpP1*, *petB*, *petD*, *rpl16*, *ndhB*, and *ndhA*. The two copies of *ndhB* are located in the inverted-repeat regions. Black and white boxes represent exons and introns, respectively, and arrowheads indicate transcriptional orientation. (b) Genomic organization and trans-splicing pattern of *rps12*. Exon 1 is located in the large single-copy region, whereas exons 2 and 3 are duplicated in IRa and IRb. Exon 1 is trans-spliced to exons 2 and 3 from either inverted-repeat copy, forming two equivalent transcript configurations. Grey, white, and black boxes represent exon 1, the IRa copies of exons 2–3, and the IRb copies of exons 2–3, respectively. Numbers below the gene models indicate their coordinates in the plastome.

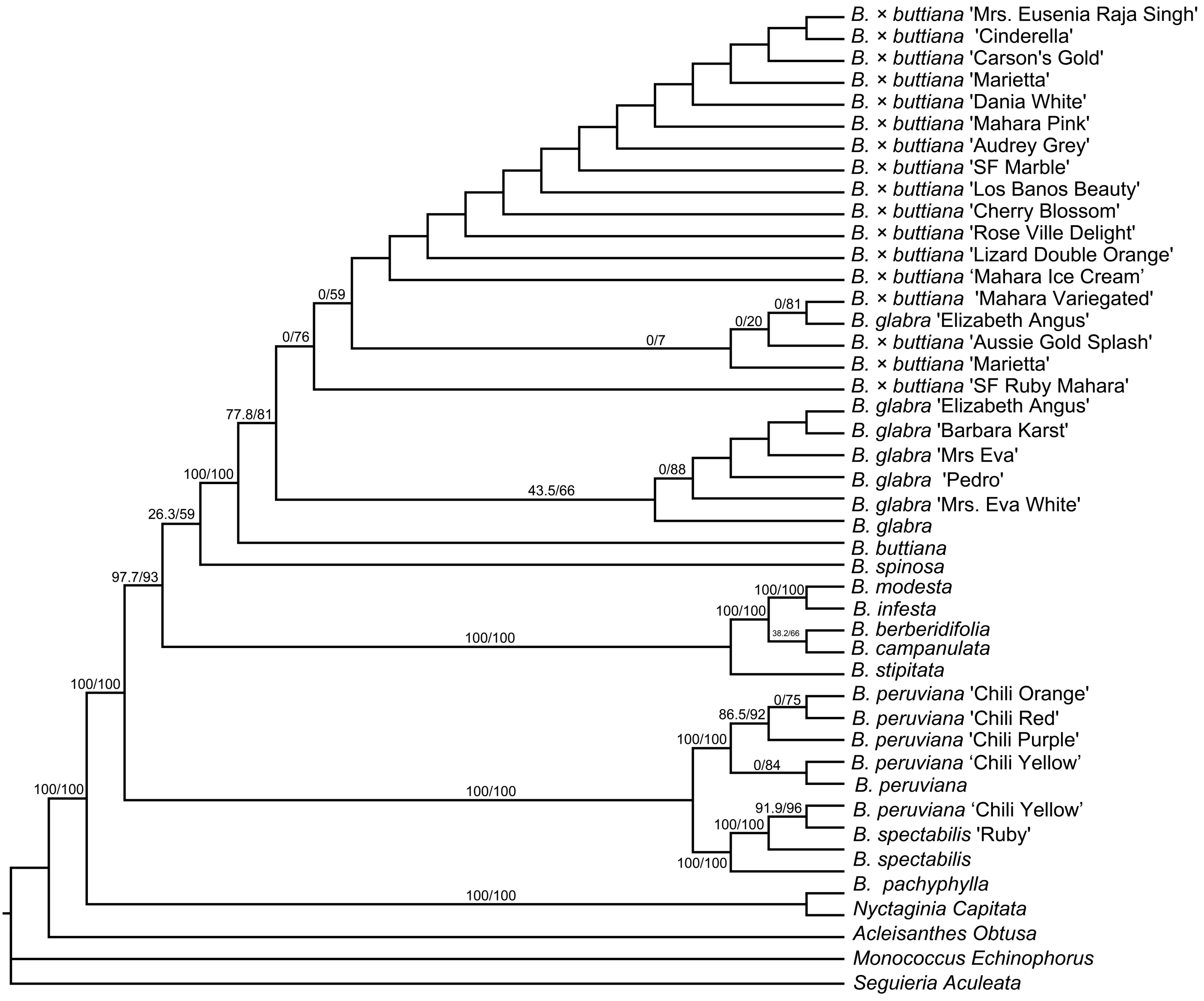

Fig. S9. Maximum-likelihood phylogeny inferred from the concatenated amino-acid sequences of plastid protein-coding genes. The maximum-likelihood (ML) tree was reconstructed from the untrimmed amino-acid alignment using IQ-TREE. Values at the nodes are presented as SH-aLRT support (%)/ultrafast bootstrap support (UFBoot, %). Although several deeper relationships received high support, many shallow nodes, particularly those among closely related *Bougainvillea* accessions, showed low support or remained unresolved. This limited resolution likely reflects the high conservation of plastid protein sequences and the resulting scarcity of phylogenetically informative amino-acid substitutions. Therefore, relationships among closely related accessions inferred from this dataset should be interpreted with caution and were not treated as the primary phylogenetic evidence.

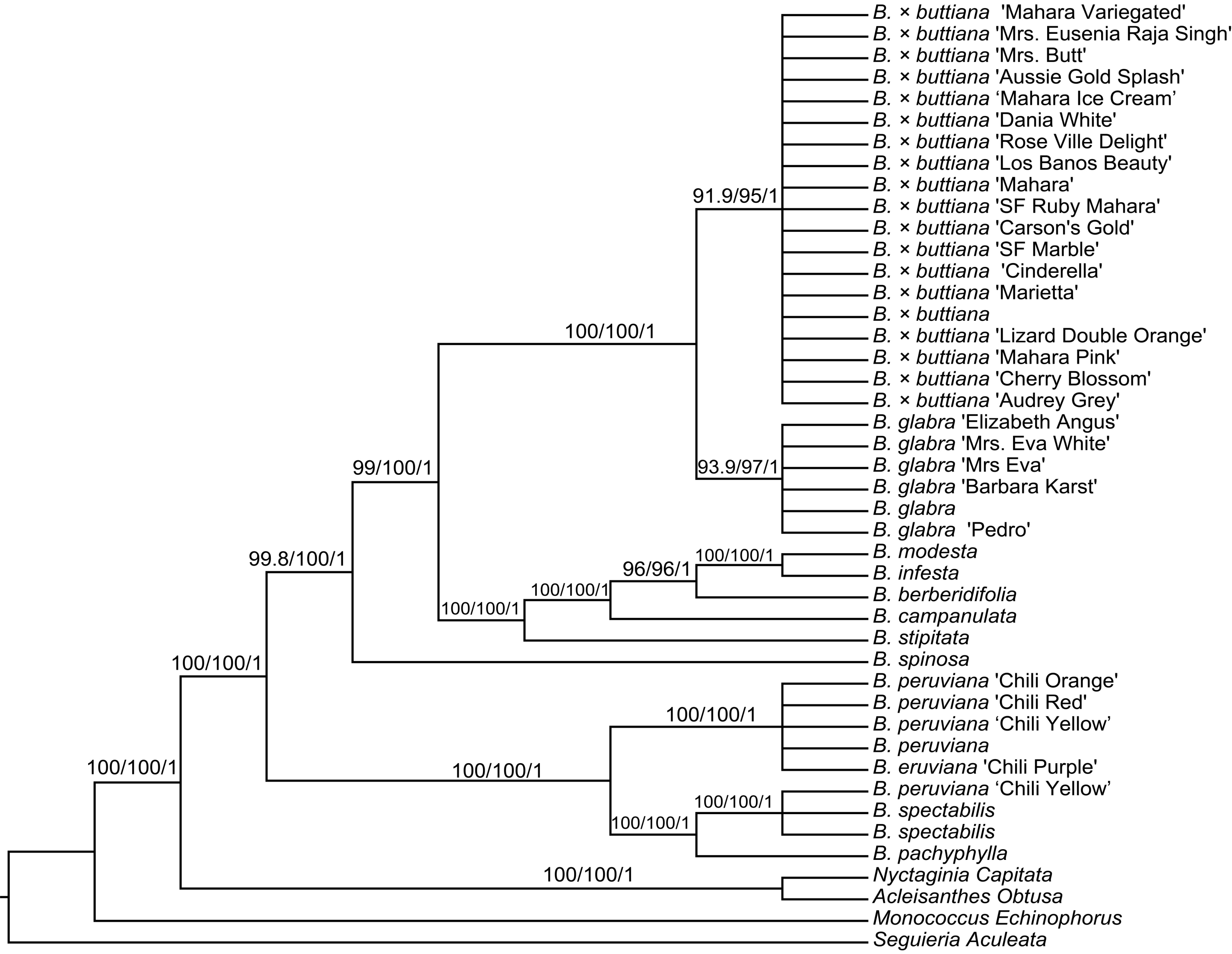

Fig. S10. Phylogenetic relationships inferred from the trimAl-trimmed complete-plastome alignment of 44 taxa. The complete-plastome sequence alignment was trimmed using trimAl to remove poorly aligned regions. Maximum-likelihood (ML) analysis was performed using IQ-TREE, and Bayesian inference (BI) was conducted using MrBayes. The ML topology is shown, with node-support values presented in the order SH-aLRT support (%)/ultrafast bootstrap support (UFBoot, %)/Bayesian posterior probability (PP). *Nyctaginia capitata*, *Acleisanthes obtusa*, *Monococcus echinophorus*, and *Seguieria aculeata* were used as outgroups. Most major nodes received high support in both the ML and BI analyses.

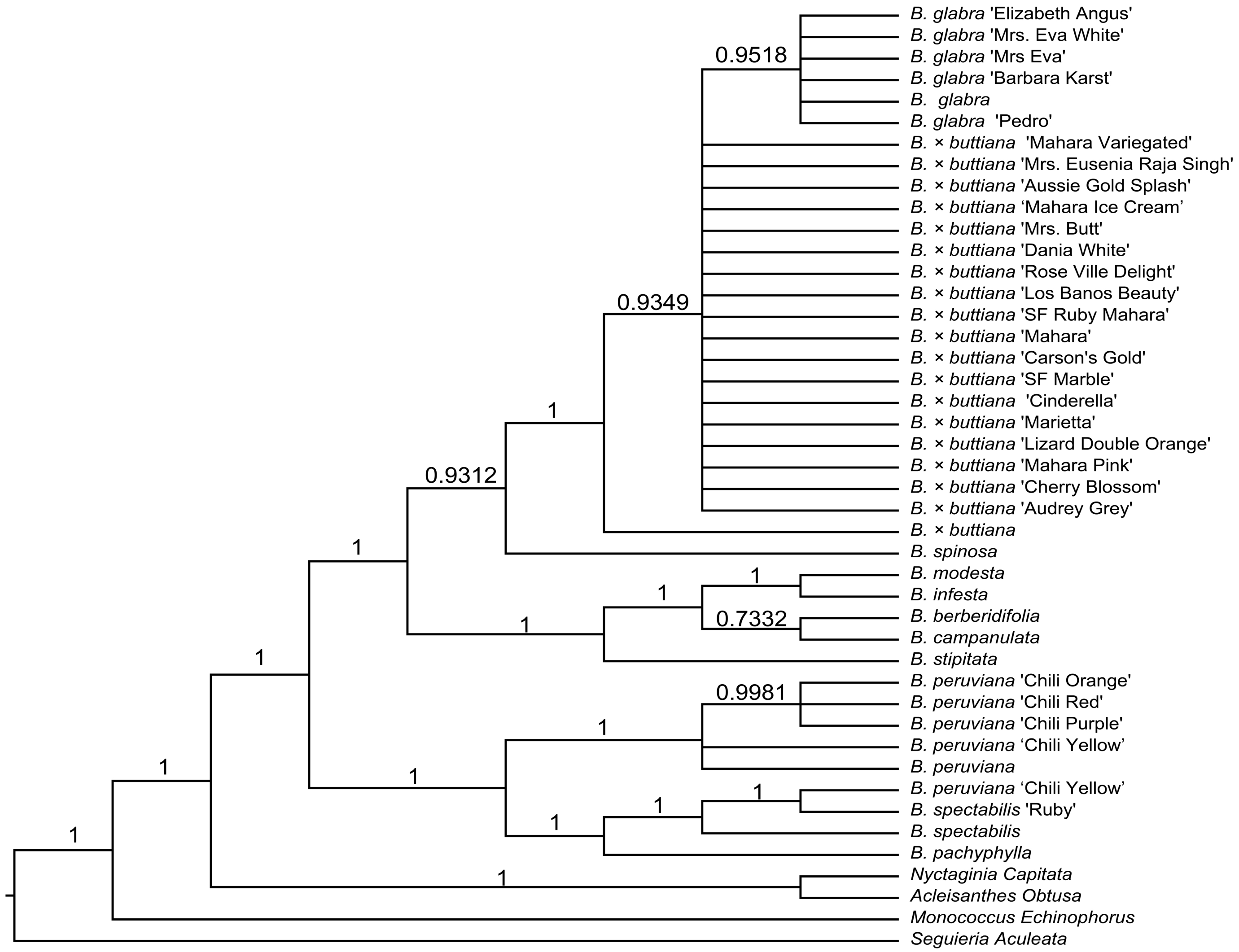

Fig. S11. Bayesian phylogeny inferred from the untrimmed concatenated amino-acid sequences of plastid protein-coding genes. Bayesian inference (BI) was performed using MrBayes based on the untrimmed concatenated amino-acid alignment. Values at the nodes represent Bayesian posterior probabilities. Although the principal deep lineages were generally well supported, several relationships among closely related *Bougainvillea* accessions received low posterior probabilities or remained unresolved as polytomies. This limited resolution likely reflects the high conservation of plastid protein sequences and the scarcity of phylogenetically informative amino-acid substitutions. Consequently, the shallow relationships inferred from this dataset should be interpreted cautiously and were not considered reliable evidence for resolving relationships among closely related cultivars.

A

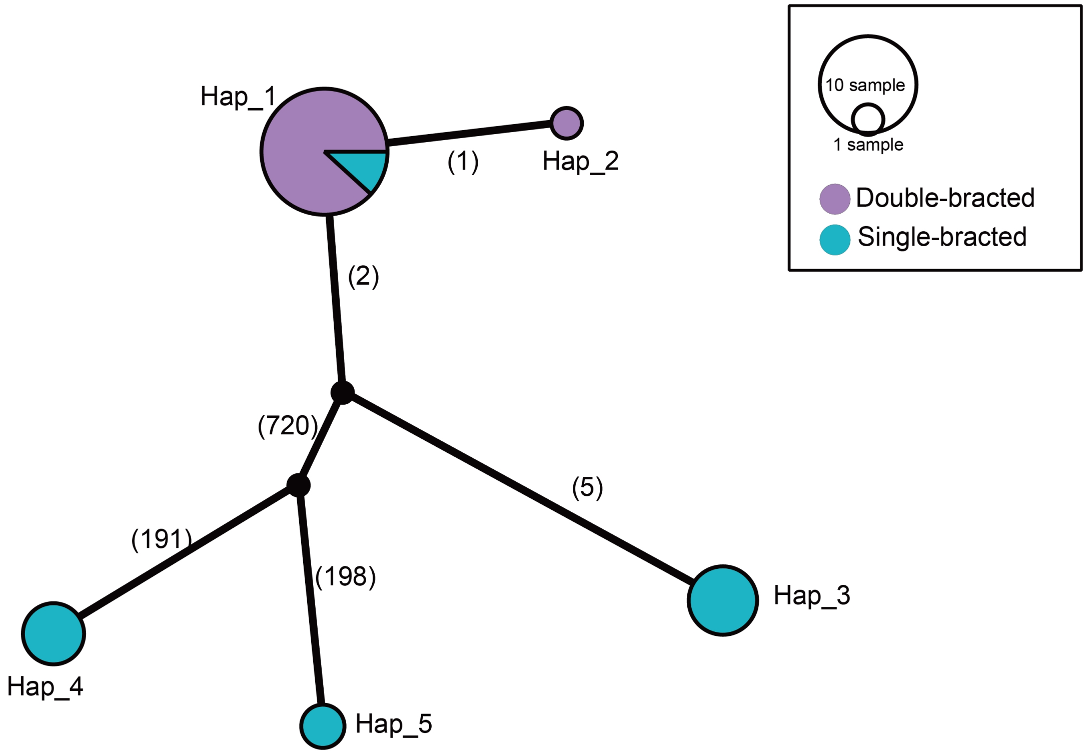

B

Sample/Hap	Hap_1	Hap_2	Hap_3	Hap_4	Hap_5
	B. × *buttiana* 'Mahara Variegated'	B. × buttiana 'Mahara'	*B. glabra* 'Elizabeth Angus'	*B. peruviana* 'Chili Orange'	*B. spectabilis* 'Flame'
	B. × *buttiana* 'Mrs. Eusenia Raja Singh'		*B. glabra* 'Mrs .Eva White'	*B. peruviana* 'Chili Red'	*B. spectabilis* 'Ruby'
	B. × *buttiana* 'Mrs. Butt'		*B. glabra* 'Mrs Eva'	*B. peruviana* 'Chili Yellow'	
	B. × *buttiana* 'Aussie Gold Splash'		*B. glabra* 'Barbara Karst'	*B. peruviana* 'Chili Purple'	
	B. × *buttiana* 'Mahara Ice Cream'		*B. glabra* 'Pedro'		
	B. × *buttiana* 'Rose Ville Delight'				
	B. × buttiana 'Los Banos Beauty'				
	B. × *buttiana* 'Carson's Gold'				
	B. × *buttiana 'SF Marble'*				
	B. × *buttiana* 'Cinderella'				
	B. × *buttiana* 'Lizard Double Orange'				
	B. × *buttiana* 'Mahara Pink'				
	B. × *buttiana* 'Cherry Blossom'				
	B. × *buttiana* 'Audrey Grey'				
	B. × *buttiana* 'SF Ruby Mahara'				
	B. × *buttiana* 'Marietta'				
	B. × *buttiana* 'Dania White'				

Fig. S12. Plastome haplotype relationships among 29 *Bougainvillea* accessions. (A) Haplotype network inferred from complete plastome sequences. Each circle represents a distinct plastome haplotype, and circle size is proportional to the number of accessions possessing that haplotype. Purple and cyan sectors represent double-bracted and single-bracted accessions, respectively. Lines connecting haplotypes indicate their mutational relationships, and numbers in parentheses indicate the numbers of mutational steps between connected haplotypes. Small black circles represent inferred intermediate haplotypes that were not observed among the sampled accessions. (B) Assignment of the 29 accessions to the five identified plastome haplotypes. Hap_1 was the predominant haplotype and was shared by both double- and single-bracted accessions, whereas the remaining haplotypes showed more restricted distributions.

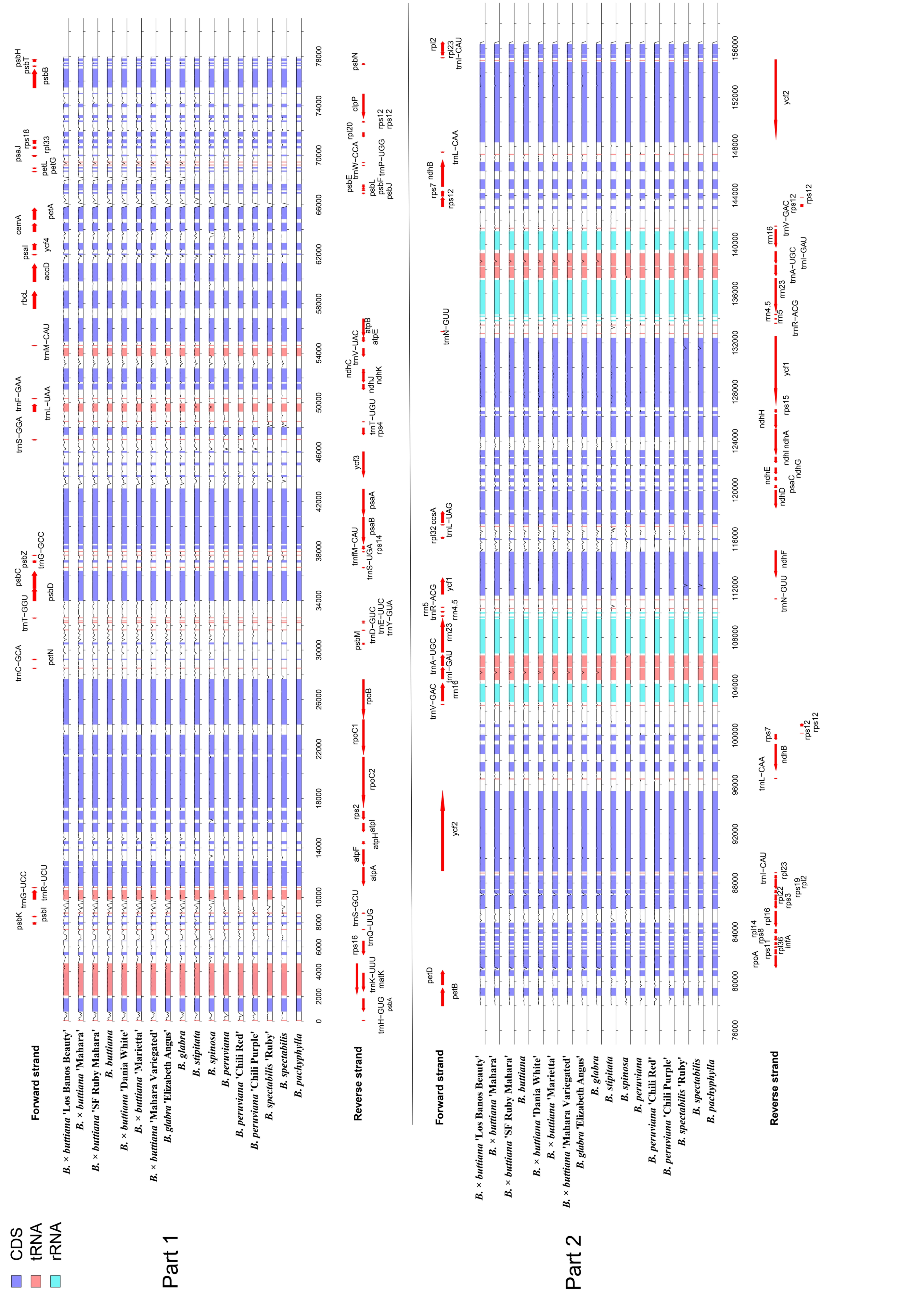

Fig. S13. Global sequence-identity comparison of 17 *Bougainvillea* plastomes with 'Los Banos Beauty' as reference (mVISTA). Red arrows indicate transcription direction; purple and white blocks denote coding and non-coding regions, respectively.

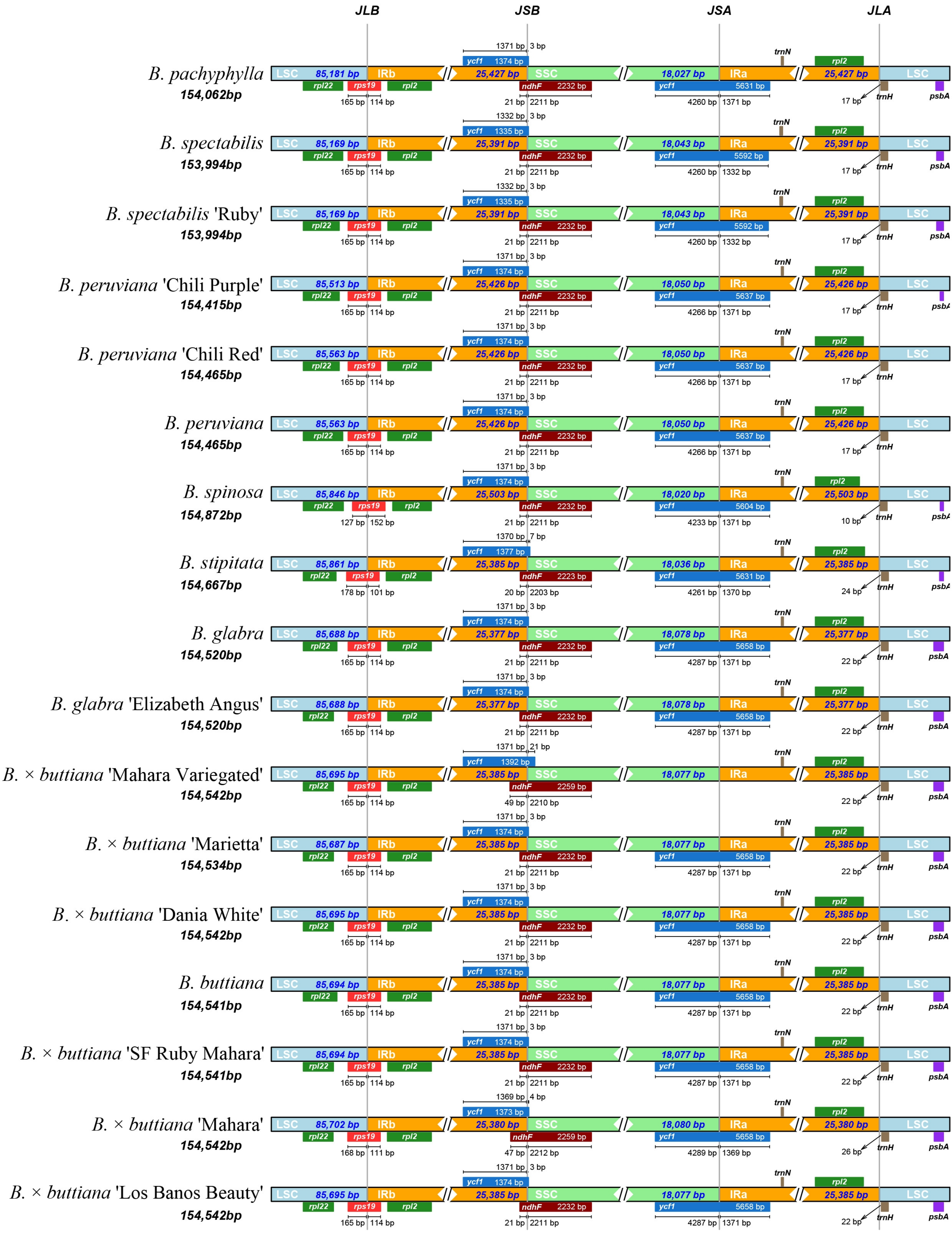

Fig S14. Comparison of the LSC/IR and SSC/IR junction regions among the 17 representative *Bougainvillea* plastomes.
